# Investigating the domestication and early management of reindeer (*Rangifer tarandus*) in the Sámi archaeological context from teeth geometric morphometrics

**DOI:** 10.1038/s41598-023-33422-6

**Published:** 2023-04-15

**Authors:** Maxime Pelletier, Emmanuel Discamps, Olivier Bignon-Lau, Anna-Kaisa Salmi

**Affiliations:** 1 Archaeology, History, Culture and Communication Studies, Faculty of Humanities, University of Oulu, Oulu, Finland; 2 CNRS UMR5608 TRACES, University of Toulouse Jean Jaurès, Toulouse, France; 3 UMR 8068 TEMPS, MSH-Mondes, Nanterre, France

**Keywords:** Palaeontology, Archaeology

## Abstract

For centuries, reindeer herding has been an integral part of the subsistence, lifeways, economy and cosmology of the Sámi of northern Fennoscandia. Despite its importance, the timing and details of early reindeer domestication are still highly debated. Identifying domesticated individuals in the archaeological record remains complicated due to the presence of two interbreeding subspecies in Fennoscandia and a mixed socio-economic organisation by Sámi populations, which was mainly a combination of wild reindeer hunting and small-scale reindeer herding. This study proposes methodological improvement for identifying domestic individuals using 2D landmark and sliding semi-landmark based geometric morphometrics on the isolated lower molars of 389 modern specimens, and 90 teeth from four archaeological sites in Finnish Lapland. Our results indicate that despite the significant impact of wear on overall tooth morphology, our protocol is very useful for identifying subspecies (classification accuracy of the two species is between 78 and 91% depending on the wear class) and understanding the morphological changes induced by the domestication process. We suggest that the morphological variation observable among modern populations has been impacted by recent changes in herding strategies in northern Fennoscandia, and that the archaeological domesticated reindeer populations were relatively different, probably due to selection by the Sámi. This study also highlights the importance of using other direct evidence or contextual archaeological data to better trace the early evidence of a domesticated reindeer economy in northern Fennoscandia, and aid in reconstructing the socio-economic changes in Sámi populations over time.

## Introduction

The domestication of reindeer (*Rangifer tarandus* Linnaeus, 1758) had a considerable impact on the lifeways, subsistence, economy and cosmology of the numerous indigenous peoples of northern Eurasia. Nowadays, reindeer herding is practiced by nearly thirty indigenous reindeer herder groups, from northern Fennoscandia to northern Mongolia and eastern Siberia^1^, resulting in most Eurasian reindeer populations now being considered domesticated or semi-domesticated^2^. Although reindeer herding constituted a key stage in the history and development of many Arctic peoples, the origin of the earliest centres of domestication and the nature of the early reindeer management strategies have not been clearly identified. Previous genetic data suggested the presence of two main independent poles of reindeer domestication, in northern Fennoscandia and Western Russia^3,4^. However, a major genetic change during the sixteenth and seventeenth centuries shows that non-native reindeer were introduced to northern Fennoscandia during this period, suggesting that the maternal lineage of modern domestic reindeer herds in Fennoscandia originated in Siberia^5^.

The earliest material evidence of reindeer management—such as sled runners or harness parts—has been found in Siberia and has been dated to ca. 1500 BC^6^ and ca. 200 BC–160 AD^7^. However, this kind of direct archaeological evidence is lacking in Fennoscandia during the early stages of reindeer domestication. In Fennoscandia, it is generally accepted that reindeer herding by the indigenous Sámi people developed gradually from the Late Iron Age onwards (ca. 800–900 AD), before becoming the main source of livelihood and the basis of social organisation ca. 1400–1600 AD^8,9^. This transition, driven by various socio-economic factors, marked a profound change in Sámi societies^9,10^. However, there was considerable geographic variation in the timing of the adoption and intensification of reindeer herding^11–13^, meaning no abrupt change from hunting to pastoralism could be identified and, making it de facto difficult to identify the beginnings of reindeer domestication from the archaeological record.

Identifying domesticated individuals in archaeological sites is all the more difficult as domestic and wild herds have widely coexisted in Fennoscandia over the last millennium. Prior to the transition to a reindeer-herding culture (i.e. before the seventeenth century), Sámi groups have long maintained their nomadic hunting traditions, while practicing small-scale reindeer herding. Indeed, they owned only small groups of domesticated individuals as decoys for hunting wild reindeer. Some male reindeer also performed various domestic tasks—such as pulling sleds and carrying freight—and females could be used for milking^11,14,15^. These particular individuals had to be tamer and kept close to settlements under fairly close supervision by Sámi herders, rather than corralled. Thus, it was also likely that Sámi herders deliberately allowed wild reindeer to crossbreed with domestic individuals in order to avoid consanguinity^12^.

**Figure 1:**
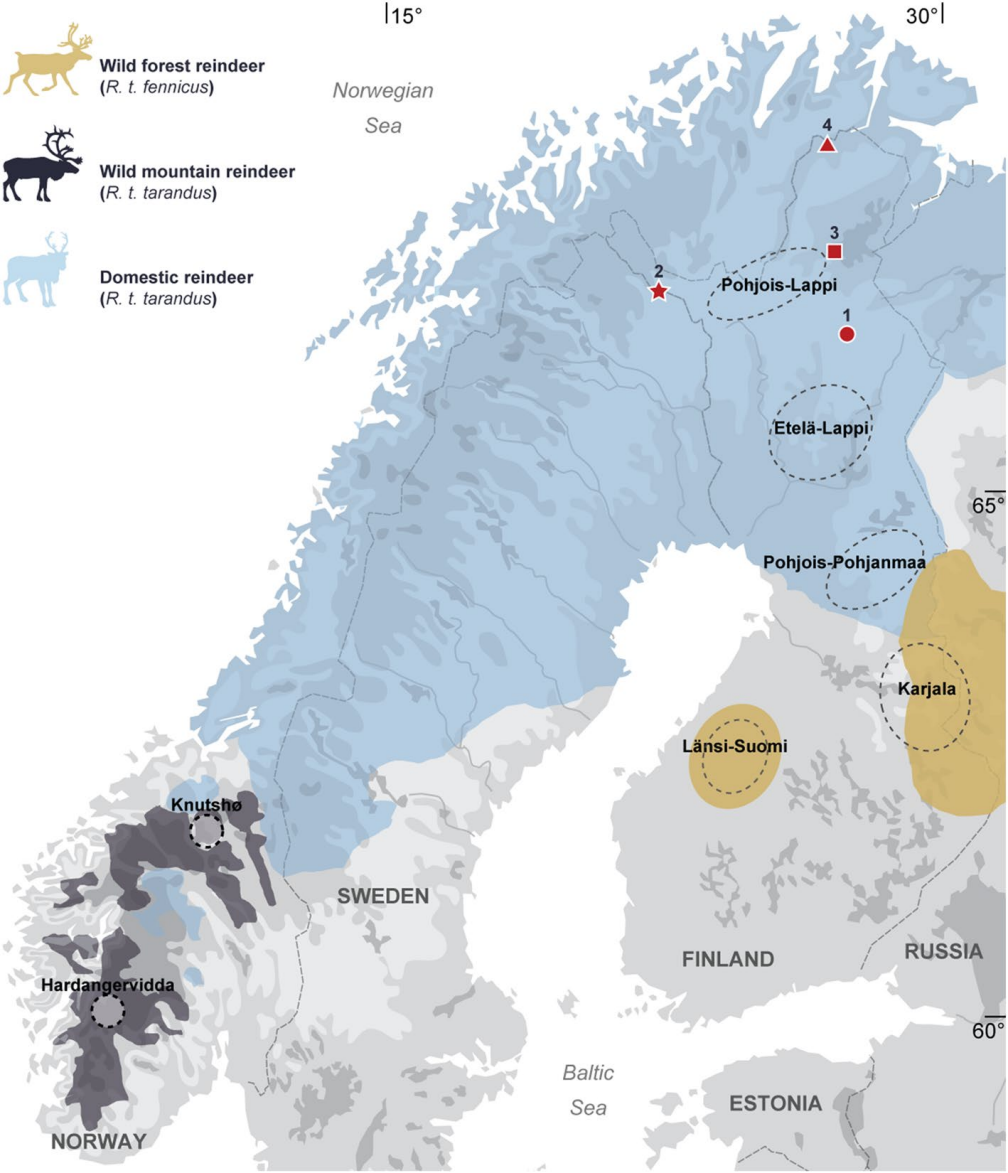
Current geographical distribution of the two reindeer subspecies^19^, including wild and domestic populations in Fennoscandia, with location of the modern populations (wild mountain reindeer: Hardangervidda and Knutshø; wild forest reindeer: Länsi-Suomi and Karjala; domesticated reindeer: Pohjois-Pohjanmaa, Etelä-Lappi and Pohjois-Lappi) and archaeological sites (1: Juikenttä; 2: Markkina; 3: Nukkumajoki; 4: Pappila) analysed.

In addition to the different reindeer husbandry and hunting strategies of the Sámi, Fennoscandia is home to two interbreeding reindeer subspecies: (1) mountain reindeer (*R. t. tarandus*), mainly inhabiting the mountainous regions of southern Norway; and (2) forest reindeer (*R. t. fennicus*), which prefer the denser habitats of the taiga in Southern Finland (Fig. 1). Fennoscandian domestic reindeer were domesticated from wild mountain reindeer^3^ and are now distributed throughout Lapland. However, the geographical distribution of these different populations and their respective biotopes has been significantly different in the past compared to the present^16^. Previously, wild mountain reindeer were found in the mountainous regions of northern Fennoscandia and wild forest reindeer were found throughout the taiga zone of Northern Finland^16^. Thus, Sámi reindeer herding is more likely to have originated in the mountainous regions of Scandinavia than in North-Eastern Fennoscandia^8,17,18^. In addition, the distribution area of the domestic herds overlapped quite extensively with the natural distribution of the wild populations, which is hardly observable nowadays.

Reindeer teeth and bones collected from archaeological sites can be a useful way of documenting the distribution range of wild reindeer subspecies before the beginning of domestication and identifying genotypic and phenotypic changes induced by the domestication process. In addition to genetic analyses using ancient DNA^5,18^ or geochemical analyses using stable isotopes^20,21^, morphometric analyses of fossil remains provide a unique opportunity to study the reindeer taxonomy and domestication process. Thus far, the morphological markers used to identify reindeer subspecies have relied on the specific characteristics of cranial elements or linear measurements of the postcranial skeleton of modern specimens^22–24^. However, these methods are difficult to apply to the archaeological record because bone remains were often broken to extract the marrow. Body-size reduction, regularly used as a domestication syndrome^25–27^, has also been observed between domestic and wild reindeer^24,28–30^. However, the presence of strong sexual dimorphism in both subspecies and a significant phenotypic plasticity can result in significant overlaps. The difficulty in disentangling the impact of human domestication from other biotic and abiotic factors^31^ makes body size a poor proxy for identifying the early stages of domestication^32,33^. Considering these biases, traditional methodologies using bone measurements or cranial morphology often fail to provide robust identification of archaeological individuals.

In order to overcome this problem, we applied geometric morphometrics which is a powerful quantitative approach that is regularly used in zooarchaeology and palaeontology to capture and graphically visualise the complexity of biological forms^34^. In recent years, these approaches have allowed a significant advance in the taxonomic identification of wild and domestic populations as well as their geographical origins in the archaeological record among several domestic ungulate species such as pig^35^, horse^36^, cattle^37^ or caprine^38^. However, only a few studies have used geometric morphometrics on reindeer remains, although they were exclusively based on modern specimens and only on postcranial skeletal bones^28–30^. Teeth are often used in geometric morphometric studies because they carry a taxonomic signal, are generally well preserved and are found in abundance in archaeological sites. Thus, such an approach has the potential to help in the subspecies identification of isolated reindeer teeth, but also in distinguishing between wild and domestic individuals in the archaeological record. For this purpose, we chose to test these biosystemic signals by studying the morphology of the enamel-dentine junction, using a 2D landmark and semi-landmark protocol applied to photographs of tooth occlusal surfaces. First and second molars (m1 and m2) were selected, considering that these teeth have similar conformation and are very well represented in archaeological assemblages. The first objective of this study was to test whether a reliable method could be devised to identify subspecies and/or the status (i.e. domestic reindeer, wild mountain reindeer and wild forest reindeer) of reindeer based on the morphology of their teeth, by documenting the morphometric diversity of seven modern populations in Fennoscandia. As a prerequisite to such an objective, we quantified the impact of sexual dimorphism and tooth wear on the morphometric variation of teeth. We then attempted to identify reindeer teeth at the subspecies level from four archaeological sites dated between the thirteenth and nineteenth centuries in order to test the method on fossil material, and thus examine the earliest archaeological evidence of a domesticated reindeer economy among the Sámi of northern Fennoscandia.

## Results

### Anatomical distinction of lower molars and wear impact

The factorial MANOVA found significant differences in shape among the two lower molar and the four wear classes investigated (Supplementary Table S1). Although the wear signal was stronger than the anatomical signal, with 47.5% against 10.2% of shape variation explained, respectively, significant interaction between both factors was found to explain 1.3% of the overall variation. This indicates that the wear varies slightly across the two teeth and therefore a separate investigation of the taxonomic resolution for each tooth is required. In addition, the discriminant model (see Supplementary Figure S4 and Supplementary Text 5.1 for more details) found correct molar identification for more than 90% of the classifications after cross-validation. This shows that this model can accurately distinguish an isolated lower molar from the archaeological record, regardless of the stage of wear.

For m1, pairwise comparisons revealed significant differences in size and shape between all wear classes (Supplementary Table S2). The more a tooth was worn, the more its centroid size increased (Supplementary Figure S5A). The shape variation of m1 was mainly due to wear and was expressed along the first principal component (PC1), accounting for 49.2% of the total variance (see Supplementary Figure S5B and Supplementary Text 5.1 for a fuller description). The effect of wear was also clearly visible on the morphology of m2. Pairwise comparisons also revealed significant differences in size and shape between wear classes, except between Classes 1 and 2 (Supplementary Table S2). Despite the overlaps, the patterns of variation seemed to evolve in a similar way to m1 (Supplementary Figure S5D-E). The centroid size increased with wear and the shape variation due to wear was also mainly expressed along the first principal component (PC1; see Supplementary Text 5.1 for a fuller description). For form analysis, the cross-validation percentage reached 71.3% for m1 and 75.6% for m2. For both teeth, the wear signal was always the largest of the shape variation explained (m1 = 55.3%; m2 = 33.4%) compared to population (m1 = 3.4%; m2 = 5.5%) or sex (m1 = 1.1%; m2 = 0.2%) (see Supplementary Table S3).These results are fundamental as they show the major impact of wear on the size and shape of the two lower molars, thereby justifying our approach of separating the analyses by wear class to better grasp the taxonomic or population signals.

### Molars size and shape variation in modern Fennoscandian reindeer populations

The results of the ANOVA on size data reveal significant differences between almost all categories for all wear classes of both teeth (*P* < 0.05 for 27 cases out of 35 possibilities; see Supplementary Table S4 and Supplementary Text 5.2 for more details). All categories displayed the same pattern of centroid size variation among ‘subspecies’, ‘subspecies + sex’, ‘status’ and ‘status + sex’. Overall, forest reindeer (*R. t. fennicus*) are significantly larger than mountain reindeer (*R. t. tarandus*), and among mountain reindeer, wild individuals were significantly larger than domestic individuals (see Supplementary Figure S6 and Supplementary Tables S5 and S6). Although the differences were not always significant between the sexes, females tended to be slightly smaller than males within the two subspecies or the different status (i.e. domestic reindeer, wild mountain reindeer and wild forest reindeer; see Supplementary Tables S7 and S8).

**Figure 2:**
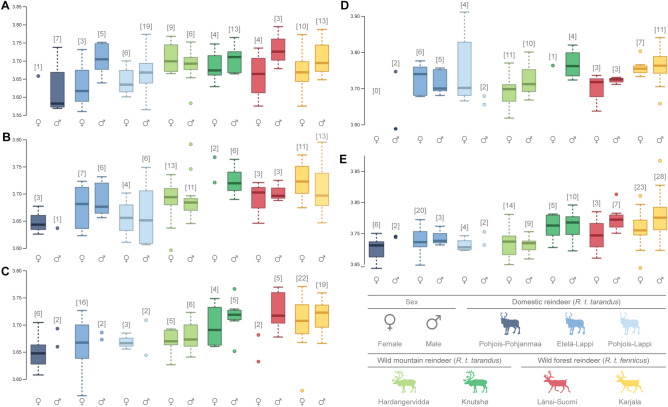
Boxplots of the variation in log-transformed centroid size (Csize) according to the population and sex for m1-Class1 (**A**), m1-Class2 (**B**), m1-Class3 (**C**), m2-Class1 (**D**) and m2-Class2 (**E**). The numbers in square brackets represent the number of teeth analysed in each population.

Although in most cases there were important overlaps in the size range of all wear classes, specificities were observed when the specimens were divided by population and sex (Fig. 2). While significant differences were not systematically found, particularly due to the small number of individuals in several of these groups, patterns of size variation appeared to be repeated across the different wear classes. Domestic populations showed a large variation in tooth size. The Pohjois-Pohjanmaa population appeared to have the smallest teeth compared to the Lapland populations (Etelä-Lappi and Pohjois-Lappi). However, the teeth of the Pohjois-Lappi population were not systematically larger than the teeth of Etelä-Lappi, indicating the absence of a latitudinal gradient in the size of modern domestic reindeer in Finland. Furthermore, sexual dimorphism appeared to be negligible within each domestic population. In wild mountain reindeer, individuals from Knutshø appeared to be slightly larger than individuals from Hardangervidda, and sexual dimorphism was also negligible. Finally, in wild Finnish forest reindeer, females tended to be smaller than males in both Länsi-Suomi and Karjala.

MANOVA analyses revealed significant differences in shape among all groups investigated between subspecies, status and populations, for both m1 and m2, and among all wear classes (Supplementary Table S9). For sex, the results were more equivocal, with no significant differences in three cases and a perceptible impact of sex on shape in the remaining 17 cases. Sex seemed to notably affect the shape of worn teeth (i.e. Class 3 of m1 and Class 2 of m2), which is consistent with previous analyses on size. The cross-validated classification rates varied quite widely depending on teeth and wear classes (Supplementary Table S9). The best classification results obtained (> 77%) concerned the subspecies and were more effective for m1 than for m2. Status (i.e. domestic reindeer, wild mountain reindeer and wild forest reindeer) generally ranked between 67 and 73% (except for Class 1 of m2, i.e. 57%). However, sex and population groups had the least effective classification results (< 60%).

**Figure 3:**
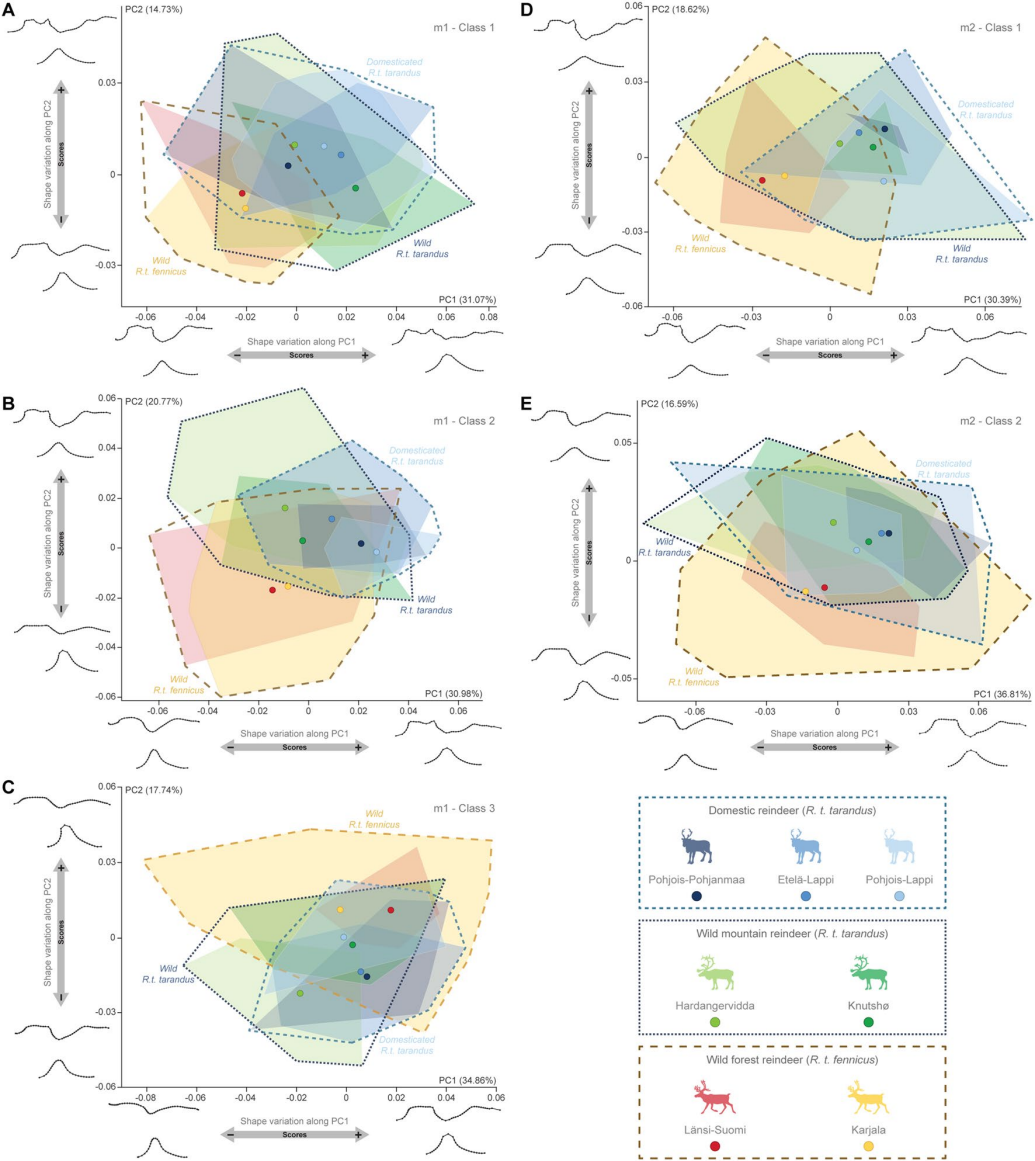
Scatter plots of the two first axes (PC1 and PC2) of principal component analyses performed on the shape data according to the population for m1-Class1 (**A**), m1-Class2 (**B**), m1-Class3 (**C**), m2-Class1 (**D**) and m2-Class2 (**E**) and visualization of shape variation via deformation of the mean shape along negative and positive PC1 and PC2 values. Each circle corresponds to the mean value of a population. The proportion of the total variance respectively expressed by the axes PC1 and PC2 is indicated in brackets.

Despite the overlaps, the patterns of variation seemed to evolve in the same way for both teeth in each wear class (Fig. 3). For Class 1 of m1 and m2, individuals showed similar variations in the morphospace: shape variation along the first principal component (PC1) revealed a subspecific variation (accounting for between 30.4 and 31.1% of shape variation depending on the tooth). For the more worn teeth (i.e. Classes 2 and 3 of m1 and Class 2 of m2), the subspecific variation was more expressed along the second principal component (PC2), accounting for between 16.6 and 20.8% of the shape variation. In contrast, for these categories, PC1 still seemed to express the shape variation for tooth wear (accounting for between 31.0 and 36.8% of the shape variation), despite the subdivision into wear classes. While the distinction between the two subspecies was relatively visible, regardless of wear class, domestic individuals and their wild mountain reindeer counterparts largely overlap in the morphospace. Ultimately, the morphological variation along the PC1 and PC2 values varied in the same pattern according to the wear classes for both m1 and m2 (see Supplementary Text 5.2).

**Figure 4:**
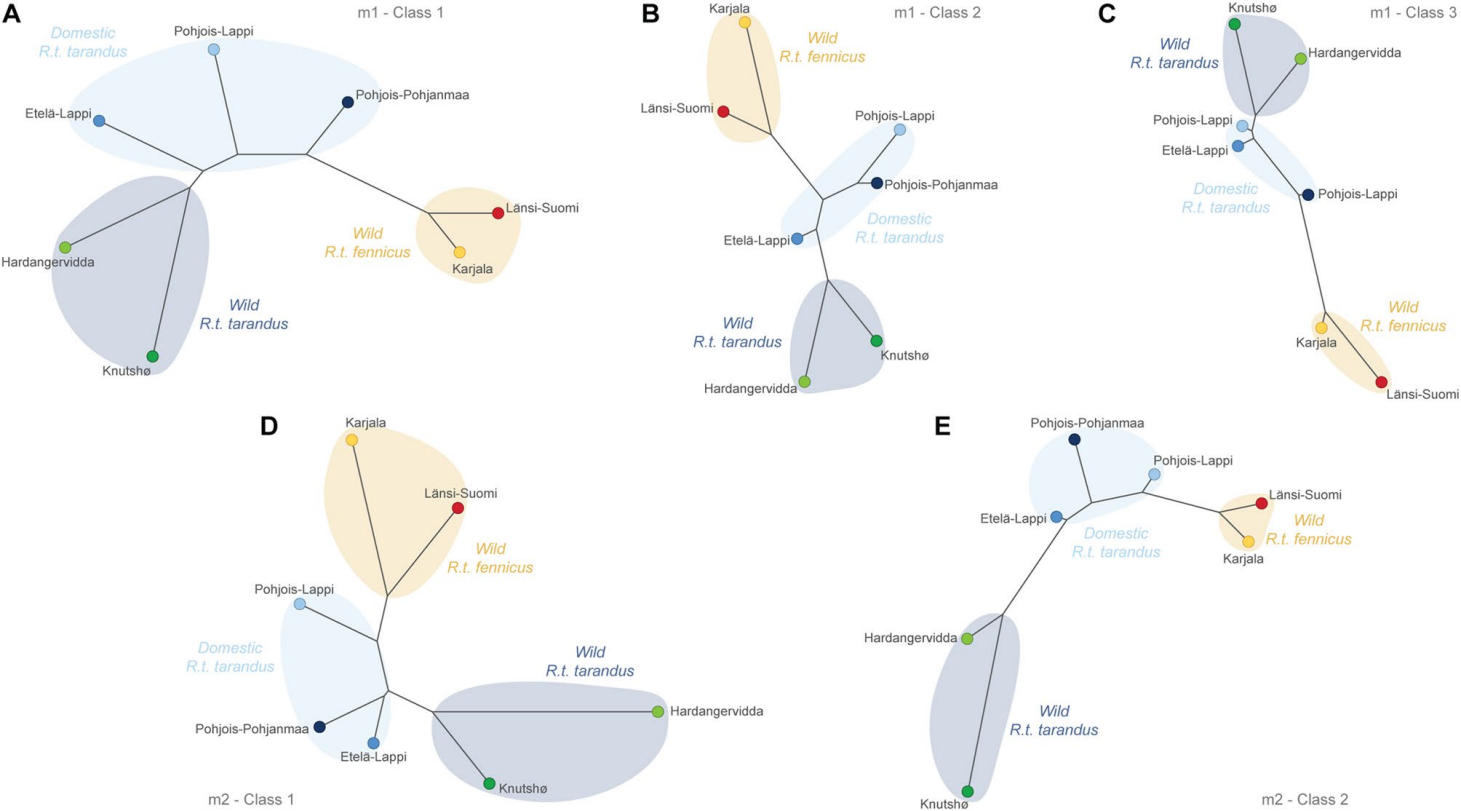
Neighbour-joining tree using Mahalanobis distances based on molar shape data from modern Fennoscandian populations for m1-Class1 (**A**), m1-Class2 (**B**), m1-Class3 (**C**), m2-Class1 (**D**) and m2-Class2 (**E**).

All five categories studied show a clear structuring of phenotypic variation for the shape (Fig. 4), where wild forest reindeer populations from Länsi-Suomi and Karjala cluster on one side of the shape networks. While the mountain reindeer populations cluster on the opposite side of the shape networks, they are clearly divided into two subgroups: the wild mountain reindeer populations from Hardangervidda and Knutshø, and the Finnish domestic populations from Pohjois-Pohjanmaa, Etelä-Lappi and Pohjois-Lappi.

For both teeth, the allometry was not significant (all *P* > 0.05) when splitting the analyses by wear class. This shows that size has no effect on shape, and vice versa. This is an essential result before any application to the archaeological record and to help in the identification of subspecies.

### Reindeer individuals from Sámi archaeological sites in northern Fennoscandia

**Figure 5:**
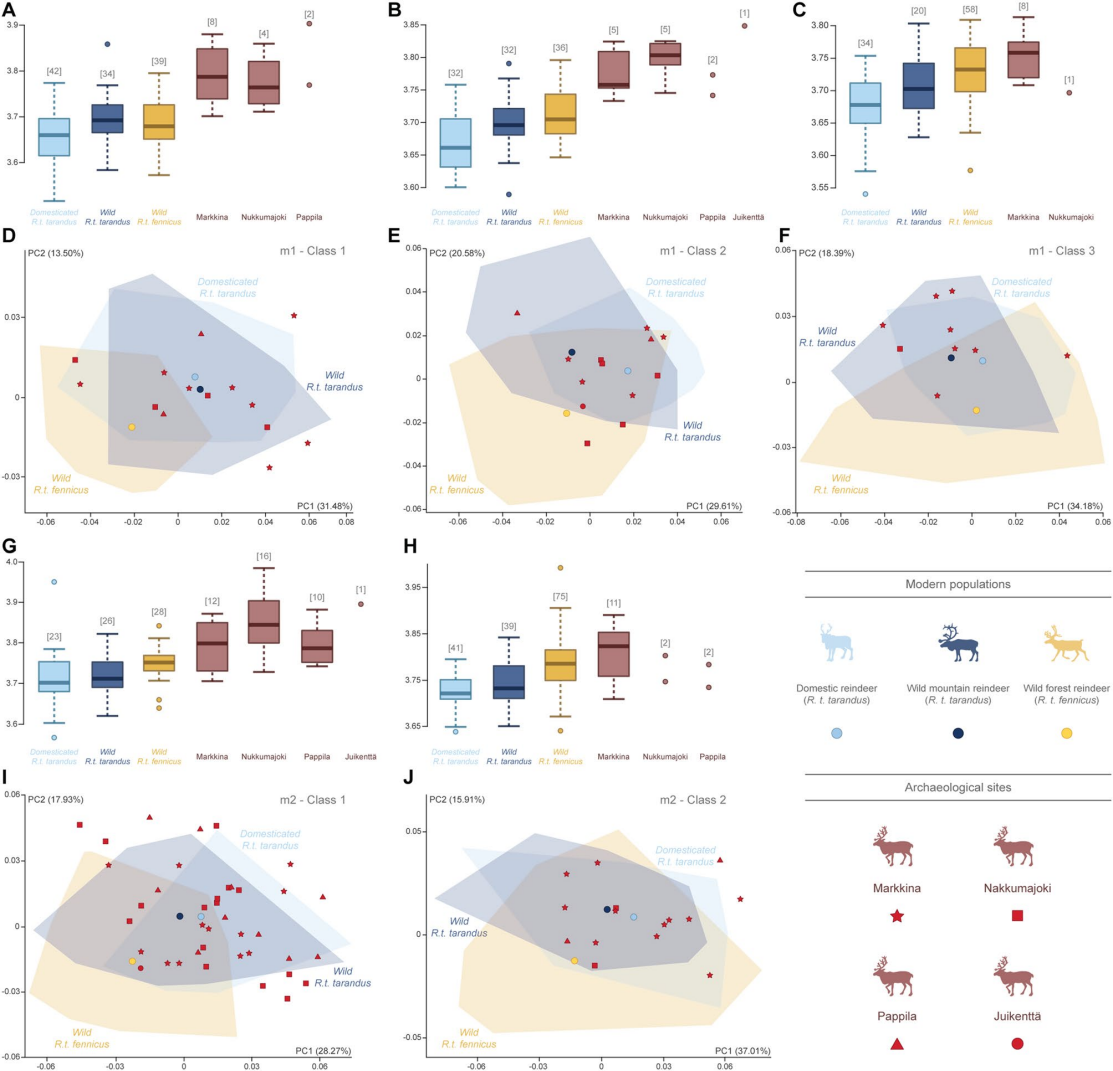
Size and shape analysis of archaeological specimens. Boxplots of the variation in log-transformed centroid size (Csize) according to the tooth wear class for m1-Class1 (**A**), m1-Class2 (**B**), m1-Class3 (**C**), m2-Class1 (**G**) and m2-Class2 (**H**). The numbers in square brackets represent the number of teeth analysed in each category. Scatter plots of the two first axes (PC1 and PC2) of principal component analyses performed on the shape data according to the tooth wear class for m1-Class1 (**D**), m1-Class2 (**E**), m1-Class3 (**F**), m2-Class1 (**I**) and m2-Class2 (**J**). The proportion of the total variance respectively expressed by the axes PC1 and PC2 is indicated in brackets.

In terms of size, the teeth of archaeological specimens were generally larger than the teeth of modern populations (all* P* < 0.05), except for Class 2 of m2 (Fig. 5A–C, G–H). In contrast, no significant differences in size were detected between reindeer from different archaeological sites (all *P* > 0.05). In terms of shape, the archaeological specimens overlapped relatively well with modern reindeer in the morphospace based on the PC1 and PC2 (Fig. 5D–F, I–J). Geometric morphometric identification of the archaeological lower molars revealed a mixed assemblage dominated by mountain reindeer (85%), followed by forest reindeer (15%). For m1, 30 teeth were attributed to mountain reindeer and six to forest reindeer. The only m1 from Juikenttä was assigned to forest reindeer. In Markkina, 19 m1 were identified as mountain reindeer (91%) and two m1 as forest reindeer (9%), and in Nukkumajoki, seven were attributed to mountain reindeer (70%) and three to forest reindeer (30%). The four m1 from Pappila have been identified as belonging to mountain reindeer. For m2, eight teeth were attributed to forest reindeer and 46 to mountain reindeer. More specifically, the only m2 from Juikenttä was assigned to forest reindeer, five teeth from Nukkumajoki were assigned to forest reindeer (against 13 mountain reindeer, 28%/ 72%) and two forest reindeer were found in faunal assemblage of Markkina (against 21 mountain reindeer, 9%/ 91%). Finally, all m2 from Pappila belong to mountain reindeer.

## Discussion

### Impact of tooth wear on geometric morphometric analyses

Despite their hardness, ungulate teeth wear with age as a result of intensive use over time and can potentially exhibit extremely variable morphology depending on the degree of wear. Recent studies on equids, bovids and caprines have indicated that age has little or no effect on the size and shape of molar occlusal surfaces^37–39^. Furthermore, these studies showed that when age-related variations in size and shape existed, they were homogeneous across species, indicating that age had a negligible impact on the distinction between taxa. However, all previous studies focused on ungulates that present significantly more hypsodont teeth, and no studies have really examined the impact of tooth wear on intra-specific morphological variation. Reindeer have brachyodont teeth whose shape is less regular along the crown. Hence, their form tends to vary much more with wear. Our study has shown that wear had a major impact on the size and shape of m1 and m2, potentially overshadowing the perceptible differences between subspecies or populations. As the tooth became worn, the centroid size of the tooth increased. Shape was also directly impacted by the wear with an attenuation of the enamel folds on the lingual side and a deepening of the interlobal grooves. Although we have been careful to subdivide our analyses into three wear classes, our results indicated that wear still played an important role in the variation of molar size and shape. Tooth wear depends on various factors such as diet, food availability, type of substrate, health status, genetics, sex or individual tooth enamel mineralisation characteristics^40–42^. However, in light of our results, we argue that the subdivision into three wear classes was a useful precaution, because even if the impact of wear remains visible, it can be taken into account in order to not misinterpret or over-interpret the results of geometric morphometric analyses.

Most of the taxonomic or population differences were more clearly evident in individuals with moderately worn teeth (i.e. Class 2). In younger individuals or in less worn teeth (i.e. Class 1), shape was more discriminating than centroid size, whereas in older individuals or in more worn teeth (i.e. Class 3), the differences were expressed more through size, and were more difficult to perceive in the morphospace. Unworn (Class 0) and highly worn (Class 4) teeth were excluded from the study, as they were difficult to integrate into our landmarking protocol, and avoided incorporating the noise inherent in these wear classes into the analyses. However, very young (i.e. Class 0) or old animals (i.e. Class 4) are generally not the most abundant individuals in the Fennoscandian archaeological record^24,43,44^. Indeed, in traditional Sámi reindeer herding, adult reindeer—mostly corresponding to our Class 2 for m1 and Classes 1 and 2 for m2—were preferentially slaughtered for meat production^45–47^. The use of young reindeer (i.e. calves) as slaughter animals only developed in Fennoscandia in the 1960s and 1970s^48,49^. The fact that there are more adult individuals in the archaeological assemblage may also be attributable to the selective hunting by Sámi societies^45^. Thus, our results from the wear classes seem to be an advantage for application to the Sámi archaeological contexts of northern Fennoscandia as these would be the best represented individuals.

### Sexual dimorphism and the influence of herd management

Part of the morphological variation between groups could be associated with the sex of the individuals—due to the existence of a well-known strong sexual dimorphism in reindeer—as already shown in geometric morphometric studies on the postcranial skeleton^28–30^. This marked biological sexual distinction can be partially explained by the spatial segregation of males and females during part of the year, but it is also accentuated by the fact that males must have a competitive advantage in competing for access to sexual partners during the rutting season^50^. Furthermore, this could directly impact teeth as there may be differences in feeding habits between males and females^51^, and a differential tooth eruption age^52^ or wear between the sexes^53^. This could explain why in our study, shape differences between males and females were only observed in individuals with worn teeth (i.e. Class 3 of m1 and Class 2 of m2). In contrast, our results indicate that males tended to have larger teeth than females, which would be consistent with the osteometric data obtained on the postcranial skeleton^24,54^. However, this was not significant in all study populations. This was slightly more evident in wild populations—especially in Finnish forest reindeer—while in domestic populations the difference was not clear. This would indicate that sexual dimorphism would have a differential impact according to the populations considered, but also that this impact is clearly less evident in terms of the size of the reindeer teeth compared to the postcranial skeleton^28–30^. Indeed, the morphological differences between sexes on bones are probably due to the different weightbearing functions of the skeletal elements. The long bones carry a greater proportion of body weight, but also the weight of antlers, which are larger and heavier in males. This biological characteristic further accentuates the sexual dimorphism, making the long bones of the limbs of reindeer much better sex discriminators than their teeth. This could also be explained by the fact that sexual selection in polygynous male ungulates favours body size over tooth size^55^.

The fact that sexual dimorphism is more noticeable in wild than in domestic populations is something that has already been observed in Finnish reindeer populations^28–30^. A more pronounced sexual dimorphism could be partially explained by a differential sex ratio. Indeed, in many polygynous wild ungulates, in which competition between males for access to females is intense and males may be at a disadvantage when competing for rare resources, the sex ratio in herds becomes biased in favour of females^56^. In domestic herds, the sex ratio can also be strongly influenced by human management and selection, which aims to maximise meat production, further favouring a sex ratio towards females^57^. Thus, as the sex ratio in domestic populations increases even more in favour of females, mating competition and the intensity of selection are likely to decrease^56^, with the potential consequence of attenuating size sexual dimorphism. Ultimately, competition is more pronounced in wild herds, so dimorphism may be more pronounced than in domestic herds.

Historically, in traditional Sámi reindeer herding, the slaughter pattern concentrated on adult males, so the proportion of males in the herds was relatively high^45,58,59^. Furthermore, the reindeer were left to graze on natural pastures as wild individuals, and their mating was probably not controlled^47^. However, the composition, structure and management of herds have changed over time, especially in the transition to reindeer herding based on more efficient meat production, with the highest possible proportion of breeding-age female reindeer^48,49,59^. This relatively recent change (i.e. from the 1960s and 1970s onwards) has affected the natural behaviour of modern reindeer herds, as well as their reproduction dynamics and genetic composition^59^. Moreover, it is also known that the body size of reindeer can be immediately influenced by human selection, without long domestic ancestry^28–30^. Thus, the relatively smaller size of modern domestic reindeer teeth compared to their wild counterparts, as well as non-significant sexual dimorphism in tooth size and shape, are more likely due to recent changes in domestic herd management. It is therefore reasonable to assume that the amplitude of sexual dimorphism must have been very different in past Sámi reindeer herds compared to what we have observed in modern domestic herds. Thus, if there are differences in the size and shape of teeth between males and females, they cannot be used to determine the sex of the animals. These data must be taken into account when applying the methodology to the archaeological record and when interpreting the results.

### Identifying Rangifer subspecies in archaeological contexts

The interpretation of fossil reindeer finds from archaeological contexts can be complicated due to the presence of two interbreeding subspecies: mountain reindeer, which include both wild and domesticated herds, as well as wild forest reindeer. Although the contact area between the two subspecies is currently limited (see Fig. 1), it was larger in the past, with mountain reindeer which was extant throughout the mountainous regions of northern Fennoscandia and forest reindeer throughout the taiga of northern Finland^16^. However, this should be assessed with the utmost caution since over time, it is historically known that the ranges of wild and domestic reindeer have fluctuated greatly under the pressure of various anthropogenic and/or climatic factors^8,60,61^. Subspecies identification is of great interest to archaeologists in terms of understanding the history of past Sámi communities since wild reindeer hunting had long been practiced in parallel with the breeding of domestic reindeer herds^10^. Taking into account that mountain reindeer have both a wild and a domestic form and that forest reindeer only have a wild form as they have never been domesticated in Fennoscandia, the identification of the subspecies seems to be a prerequisite before any identification of domestic individuals in archaeological faunal assemblages can take place. Thus, the identification of the subspecies may reflect different subsistence strategies—such as hunting or herding—and/or cultural interpretations (e.g. Sámi sacrificial offering or food scraps).

Previous studies have shown that the morphologies of these two subspecies overlap considerably, making it difficult to identify them from often fragmented archaeological bones^24^. In our study, the analyses of morphological variation in teeth brought new clues about subspecific discrimination. We showed that modern wild forest reindeer had significantly larger molars than modern mountain reindeer—both wild and domestic—which is in accordance with previous osteometric and geometric morphometric studies on the postcranial skeleton^24,28–30^. Regarding shape, our results allow also a relatively good distinction to be made between the two reindeer subspecies currently living in Fennoscandia, with more than 80% of correct classification with cross-validation (except for Class 1 of m2: 78%), which may help to distinguish between them in the fossil record. The congruence of these morphotypes with phylogeny confirmed that the phylogenetic signal on the shape of lower molar is relatively strong. The morphological differences between subspecies could also reflect different geographical and ecological origins prior to their migration to Fennoscandia. Reindeer were already present in Norway and Sweden by 13,000–12,000 BP, while their presence in Southern Finland only dates back to 7000 BP^60^. Thus, one of the main hypotheses is that mountain reindeer are the descendants of the South-European Pleistocene reindeer and migrated into northern Fennoscandia via the west coast of present-day Norway, while forest reindeer probably colonised south-eastern Fennoscandia directly from eastern Siberia or south-eastern Central Europe. Although many authors have addressed the question of the disappearance of reindeer from Southern Europe after the Last Glacial and their subsequent colonisation of Northern Europe^60,62–65^, no study has used distinctive morphology to highlight the different migration routes of these two subspecies following the melting of the ice cap. Thus, our geometric morphometric results could eventually test these hypotheses and identify the geographical origin of each subspecies.

Morphometric identification of the lower molars of fossil reindeer revealed the presence of both subspecies in the archaeological faunal assemblage of northern Fennoscandia—dominated by mountain reindeer (85%) followed by forest reindeer (15%). In Juikenttä, m1 and m2 (belonging to the same individual, included in the same hemi-mandible) were found to belong to a forest reindeer. As the site is located in the boreal forest, this confirms the presence of this subspecies in the region between the thirteenth and seventeenth centuries. However, the presence of wild forest reindeer in an archaeological assemblage is not necessarily proof of the absence of domestication by Sámi communities. Indeed, a recent study of reindeer bone remains from Juikenttä has shown the use of draught reindeer at the site from at least the late thirteenth century^66^. This shows the presence of both subspecies in the region at that time and therefore a mixed socio-economic organisation by Sámi populations shared between reindeer herding and wild reindeer hunting. Conversely, all of the individuals identified in Pappila were mountain reindeer. As the site is located in the fell region, a high and barren landscape, the environment was probably never suitable for forest reindeer. The Sámi communities practised reindeer herding in this area in the seventeenth century, but subsistence was mainly based on hunting and fishing^45^. The absence of forest reindeer in the Pappila faunal assemblage does not necessarily mean that there were only domestic reindeer at the site, but suggests that hunting strategies must have been geared towards wild mountain reindeer.

In Nukkumajoki and Markkina, sites located on the northern edge of the boreal forest, both subspecies were identified in the faunal assemblage but in different proportions. This means that both species were present in these two regions between the fifteenth and nineteenth centuries, and that Sámi subsistence patterns were also mixed (hunting/herding). Indeed, evidence for the use of draught reindeer has been found at Nukkumajoki^66^, and it is historically known that reindeer herding was already practised in the Enontekiö region when the Markkina site was occupied^44^. In Markkina, almost 91% of the analysed teeth (m1 and m2) were attributed to mountain reindeer, in contrast to forest reindeer (nearly 9%). In Nukkumajoki, the faunal assemblage is also largely dominated by mountain reindeer, but with a higher proportion of forest reindeer than in Markkina (30%). This could indicate that the population density of forest reindeer was higher in north-eastern than in north-western Finland, where the region becomes more mountainous and probably less suitable for forest reindeer than for mountain reindeer. However, a different proportion of mountain and forest reindeer at these two sites may also be the attributable to the Sámi communities' choice of hunting and subsistence strategies. Indeed, mountain reindeer are more gregarious, they live in more open tundra or mountain regions, while forest reindeer have a more solitary and complex social organisation in a more closed taiga environment^22^. Trapping pit systems, which can comprise hundreds of pitfall traps at strategic locations in the landscape, were a widespread Sámi hunting technique for wild reindeer in northern Fennoscandia during the Iron Age and medieval periods^12,67,68^. This hunting strategy was probably more suited to hunting wild mountain reindeer than wild forest reindeer. The differential proportion of the two subspecies found in archaeological sites could also reflect the function of the site. Nukkumajoki is a village, while Markkina is a marketplace. Wild forest reindeer may have been consumed more at the settlement sites than at the markets.

### Explaining size differences between modern and past reindeer teeth

In our archaeological sample, the teeth of fossil specimens were generally significantly larger than those of modern individuals. This could be partially explained by the fact that there are no longer any completely wild modern genetic lineages of mountain reindeer in northern Fennoscandia, following the introgression of domestic reindeer into the wild gene pool in the nineteenth century^4^. Furthermore, past wild populations lived at much higher latitudes than present-day wild populations. Wild mountain and forest populations are now confined to Southern Norway and Finland, respectively, but have occupied northern Fennoscandia in the past^16^, as our results also confirmed. Weinstock^69^ stated that larger reindeer were found at higher latitudes and/or in colder environments, conclusions that are in line with Bergmann's rule, although this has been questioned for present-day Canadian^70^ or Pleistocene Southern European populations^71^. The reduction in tooth size between modern domestic reindeer and the 13th to seventeenth century domestic reindeer potentially present at these archaeological sites could also be due to changes in reindeer herd management, selective breeding, and herding practices in the late twentieth century^47–49^. However, working domestic individuals have been shown to be taller than free-ranging domestic individuals, which is explained by the fact that working individuals are selected for their physical properties and abilities (usually males)^28–30^. Size reduction should therefore not be a reliable criterion for identifying domestic reindeer in the archaeological record; on the contrary, they should be significantly larger.

Thus, the fossil sample comprises wild forest reindeer that are generally larger than mountain reindeer, as well as mountain reindeer, possibly including both wild and domesticated individuals. Previous studies have shown that reindeer herding was already present during the occupation phase of the Markkina and Pappila markets^44^. In addition, the age and sex structure of reindeer in these sites would argue for the presence of domesticated individuals, including male individuals. In Juikenttä and Nukkumajoki, the use of draught reindeer has also been suggested, which are usually male individuals^66^. We therefore argue that the large tooth size of archaeological individuals is explained by the presence of forest reindeer, but also by a high proportion of male domestic mountain reindeer, which are generally larger. Although our method is very reliable in distinguishing between subspecies, the presence of mixed subsistence strategies for several centuries in northern Fennoscandia makes it relatively complicated to identify early domesticated reindeer in the archaeological record. Thus, our method should still be supported by other material evidence such as harness pieces^72^ or the presence of palaeopathologies and/or entheseal changes directly on the reindeer bones^66^.

## Conclusion

Historically, reindeer is probably one of the species to be most recently domesticated by humans, but identifying the period and place of origin of their domestication through the archaeological record remains a complex task. Thus, zooarchaeologists need powerful biomarkers on fossil reindeer remains to document the origin of early domesticated reindeer. Our work demonstrated the potential of geometric morphometric studies on the lower molars to identify subspecies, but also to better understand the impact of tooth wear and sex on the tooth morphology of different Fennoscandian reindeer populations.

Understanding the morphometric variability of reindeer had to be carried out beforehand by bringing together a large sample of modern specimens before application to the fossil record. Our results showed that tooth wear had a major impact on the size and shape of the lower molars, unlike sex, which had a negligible impact. Nevertheless, this new protocol allowed for a very reliable taxonomic distinction at the subspecific level, and also allowed for a discussion on the morphological variations between wild and domestic individuals, in modern populations and archaeological assemblages. This methodology will allow archaeologists to better estimate the presence of wild or domestic reindeer in archaeological assemblages, and thereby comprehend the evolution of socio-economic models of the Sámi reindeer herder communities in northern Fennoscandia. However, caution must be taken with regard to the correct identification of domestic reindeer due to the great variability in the timing and dispersal of the domestication process in Fennoscandia, as well as the genetic introgression between wild and domestic herds. Each variable—such as wear, sex or taxonomy—and parameter—such as size, shape and allometry—must be finely analysed and coupled with archaeological contexts in order to be able to identify individuals and better understand the morphometric variability of archaeological reindeer.

Nevertheless, the results of our analyses on archaeological individuals have confirmed the presence of a mixed economy of hunting and herding with regional variations, which could be related to ecological and/or cultural factors. The archaeological individuals considered in this study came from four North-Eastern Fennoscandia sites dated between the 13th and the eighteenth centuries, although Sámi reindeer herding might date back further, in other regions such as the Scandinavian mountains. There could even be material evidence of earlier reindeer management in Siberia, linked to other past Arctic communities. Thus, future work should include these other geo-chronological contexts in which our methodology should help to identify the oldest traces of reindeer domestication. Such studies would allow for the refinement of research on archaeological sites in order to improve the identification of the first stages of reindeer domestication in time and space. Additionally, in a broader perspective, our analytical protocol should be able to provide subspecies identification of *Rangifer* individuals for other archaeological periods, such as the rich Palaeolithic record of reindeer hunting in Eurasia through time.

## Methods

Geometric morphometrics were performed on standardised 2D images of the first (m1) and second (m2) lower molars, collected from modern and archaeological reindeer samples (Table 1). A total of 389 modern specimens (n_teeth_ = 581; n_m1_ = 327 and n_m2_ = 254) were analysed, including the two extant Fennoscandian reindeer subspecies—both wild and domestic—from seven localities (Fig. 1; Supplementary Text 1). The archaeological reindeer teeth (n_teeth_ = 90; n_m1_ = 36 and n_m2_ = 54) that were analysed came from two Sámi dwelling sites and two Sámi market places in present-day Northern Finland, dating from ca. 1300 to 1800 AD^44,66^ (Fig. 1; Supplementary Text 2).

**Table 1:** Detail of modern specimens studied from the Zoological Museum of Oulu (wild forest reindeer and domestic reindeer from Finland) and the UMR 7041 ArScAn laboratory (wild mountain reindeer from Norway) according to locality, sex (female: ♀; male: ♂) and tooth (first lower molar: m1; second lower molar: m2), as well as archaeological specimens from the Finnish Heritage Agency of Helsinki, Finland.

	Number of individuals	m1				m2			
		♀	♂	ND	Total	♀	♂	ND	Total
*Modern wild forest reindeer* (*R.t. fennicus*)									
Länsi-Suomi (Southwestern Finland)	31	9	11	7	27	7	10	4	21
Karjala (Southeastern Finland)	129	43	45	18	106	36	41	17	94
*Modern wild mountain reindeer* (*R.t. tarandus*)									
Hardangervidda (Southern Norway)	67	22	30	–	52	25	21	–	46
Knutshø (Southern Norway)	42	15	17	2	34	6	15	1	22
*Modern domestic reindeer* (*R.t. tarandus*)									
Pohjois-Pohjanmaa (Northern Ostrobothnia, Finland)	28	10	10	5	25	6	5	4	15
Etelä-Lappi (Southern Lapland, Finland)	49	26	13	2	41	29	8	2	39
Pohjois-Lappi (Northern Lapland, Finland)	43	13	27	2	42	8	7	2	17
Total	389	138	153	36	327	117	107	30	254

Archaeological samples	N teeth	m1	m2						
Juikenttä (Sodankylä, Lapland, Finland)	2	1	1						
Markkina (Enontekiö, Lapland, Finland)	44	21	23						
Nukkumajoki 2 (Inari, Lapland, Finland)	28	10	18						
Pappila (Utsjoki, Lapland, Finland)	16	4	12						
Total	90	36	54						

As tooth wear can have a significant impact on overall tooth morphology, wear classes were established to test the impact of wear on our geometric morphometric results. Four classes were used (Class 0 to Class 4, Supplementary Figure S1 and S2; see Supplementary Text 3 for a justification of this classification). For each tooth, the occlusal view was photographed using a standardised protocol (Supplementary Text 4). Modern Norwegian populations were photographed by ED and Modern Finnish modern populations and archaeological specimens were photographed by MP. Following data acquisition, MP reviewed each photograph and only those that strictly respected the established protocol were considered in the study.

**Figure 6:**
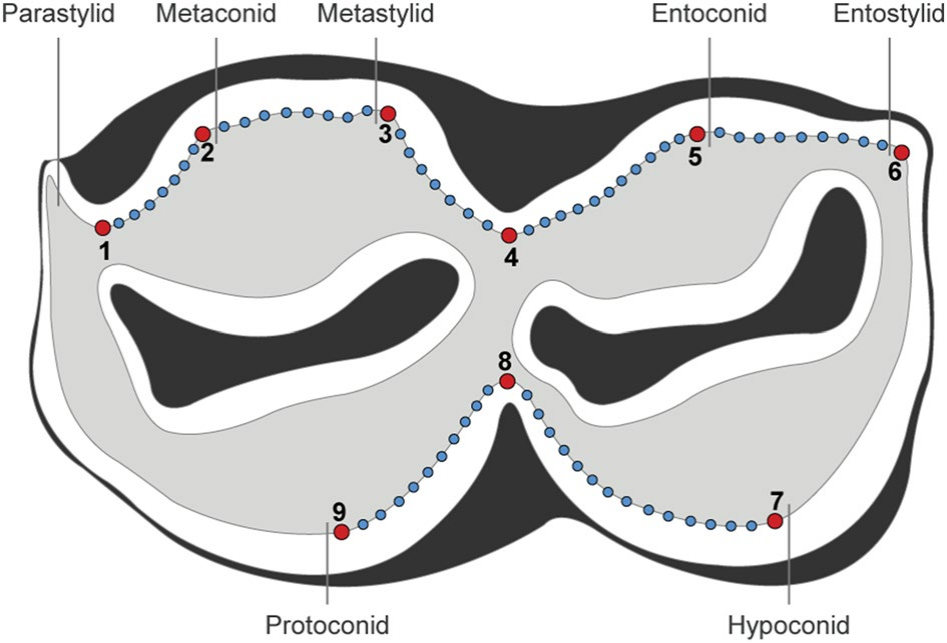
Landmarks and semilandmarks locations on the left lower molar (m1 and m2) of reindeer in occlusal view. **1**: point of maximum curvature between the parastylid and the metaconid; **2**: point of maximum curvature on the metaconid; **3**: point of maximum curvature on the metastylid; **4**: point of maximum curvature between the metastylid and the entoconid; **5**: point of maximum curvature on the entoconid; **6**: point of maximum curvature on the entostylid; **7**: point of maximum curvature on the hypoconid; **8**: point of maximum curvature between the hypoconid and the protoconid; **9**: point of maximum curvature on the protoconid.

In order to properly define the position of our landmarks and their relevance, we first quantified the number of individuals showing loss of enamel contact and exposed dentine connections according to the wear classes, (see Supplementary Figure S3 and Supplementary Text 4 for a justification of this protocol). The shape and size of m1 and m2 was estimated using a new 2D protocol involving nine landmarks and 66 equidistant sliding semilandmarks positioned on the inner edge of the enamel, i.e. at the enamel-dentine junction, to limit biases related to external enamel wear (Fig. 6 and Supplementary Text 4 for more details). The landmark and semi-landmark coordinates were acquired by a single operator (MP) from digital photographs using tpsDig2 v.2.16^73^. Teeth with little to no wear (Class 0) were excluded from the study because tooth wear rendered the landmark protocol inapplicable due to a non-visible enamel-dentin junction or minimal wear with unexposed dentinal connections. Similarly, highly worn (Class 4) teeth were excluded from the analyses, as most individuals had excessively worn teeth for which the protocol was also inapplicable due to the total or partial disappearance of the enamel-dentin junction. Particularly for m2, Class 3 was also excluded due to an insufficient number of individuals (n = 16).

Unlike landmarks, semilandmarks do not have an exact anatomical correspondence along the enamel-dentine junction, and instead ‘slide’ along the line between adjacent points in order to minimise the bending energy of the thin-plate spline interpolation function^74^. All specimens’ coordinates were aligned using the Generalized Procrustes Analysis (GPA) during which the sliding semi-landmark coordinates were allowed to slide using the bending energy criteria^75^ and conducted using tpsRelw v.1.49^76^. All configurations were translated, normalised and rotated to minimise the overall sum of the squared distances between the corresponding landmarks and semilandmarks. To remove the effects of scale, GPA also computes a unit centroid size as the square root of the summed squared distances from all landmarks and semilandmarks to their centroid^77^.

The analyses were first performed on the whole set of teeth (m1 and m2) in order to verify whether the method could easily distinguishing between the two types of molars, which have a very similar general conformation. A canonical analysis of variance (CVA), paired with a correct cross-validation test, were then performed in order to obtain classification accuracy. CVA was performed for shape. Secondly, the analyses focused on tooth wear for each molar to measure its impact on morphology. For this purpose, differences in m1 and m2 between reindeer populations and then by wear class were first visualised using boxplot for size and Principal Component Analysis (PCA) for shape. Finally, the analyses were split by tooth and by wear class to better grasp the taxonomic and/or population signal. Size differences were also visualised using a boxplot based on log-transformed centroid size and evaluated using an analysis of variance (ANOVA) with an error threshold set at α = 5%. The analyses were segmented by pooling the specimens by (1) tooth; (2) wear classes; (3) subspecies; (4) status (i.e. domestic reindeer, wild mountain reindeer and wild forest reindeer); (5) sex; and (6) populations. Pairwise comparisons of the populations were performed using multiple Wilcoxon rank tests according to these different categories. To control for the false discovery rate, a multicomparison correction was applied to the *P*-values using the ‘Benjamini-Hochberg’ method^78^. Shape differences between these different groups were estimated using a multivariate analysis of variance (MANOVA), with significant interaction (α = 5%) assumed to reflect group differences. Shape variation was visualised using a principal component analysis (PCA) based on Procrustes coordinates. We then assessed the specific and/or population assignment accuracy by calculating the cross-validated correct classification percentages, using a CVA. So as not affect the cross-validation results, we reduced the dimensionality of our data set by keeping the values of the main components expressing 95% of the total variance before each CVA^79^. The phenotypic similarities between groups were calculated from Mahalanobis distances derived from canonical variates and visualised using a neighbour-joining network^80^. Allometry was assessed using multivariate regressions of shape variables on the log-transformed centroid sizes. Finally, for the identification of archaeological reindeer, fossil individuals were superimposed along the modern populations and a linear predictive discriminant analysis was performed on the shape data. All morphometric statistics were performed with Rstudio v.1.1.383^81^, using the ‘ade4’^82^, ‘Geomorph’^83^ and ‘Morpho’^84^ libraries.

## Supplementary Information

Supplementary Information.

## Data Availability

The datasets used and/or analysed during the current study available from the corresponding author on reasonable request.
