# Supplementary material for: Investigating the domestication and early management of reindeer (Rangifer tarandus) in the Sámi archaeological context from teeth geometric morphometrics
Source: Sci Rep. 2023 Apr 15;13:6174. doi: 10.1038/s41598-023-33422-6 (PMC10105696; doi:10.1038/s41598-023-33422-6)
Supplement: Supplementary file 1 — Supplementary Information. [file 41598_2023_33422_MOESM1_ESM.pdf]

Supplementary Information for

Investigating the domestication and early management of reindeer (*Rangifer tarandus*) in the Sámi archaeological context: new insights from teeth geometric morphometrics

Maxime PELLETIER, Emmanuel DISCAMPS, Olivier BIGNON-LAU, Anna-Kaisa SALMI

Corresponding author: **Maxime PELLETIER**

Email address: *maxime.pelletier@oulu.fi*

<u>This PDF file includes</u>:

Supplementary Texts 1 to 5:

1. Modern reindeer sample (*p. 2*)
2. Archaeological site backgrounds (*p. 2-3*)
3. Age and establishment of wear classes (*p. 3-4*)
4. Tooth landmarks (*p. 5-7*)
5. Results (5.1. *p. 8-9;* 5.2. *p. 11-20*)

Supplementary Figures S1 to S7:

Figure S1 (*p. 3*)
Figure S2 (*p. 4*)
Figure S3 (*p. 6*)
Figure S4 (*p. 8*)
Figure S5 (*p. 9*)
Figure S6 (*p. 12*)
Figure S7 (*p. 21*)

Supplementary Tables S1 to S13:

Table S1 (*p. 8*)
Table S2 (*p. 9*)
Table S3 (*p. 10*)
Table S4 (*p. 11*)
Table S5 (*p. 13*)
Table S6 (*p. 13*)
Table S7 (*p. 14*)
Table S8 (*p. 15*)
Table S9 (*p. 16*)
Table S10 (*p. 16*)
Table S11 (*p. 17*)
Table S12 (*p. 18*)
Table S13 (*p. 19*)

References (*p. 22*)

1. Modern reindeer sample

A total of 389 modern specimens, including the two extant Fennoscandian reindeer subspecies, were analysed from seven localities (Figure 1): wild mountain reindeer (*R. t. tarandus*), from Hardangervidda (n = 67) and Knutshø (n = 42) in Southern Norway; wild forest reindeer (*R. t. fennicus*), mainly from Perho in southwestern Finland (Länsi-Suomi, n = 31) and Khumo in southeastern Finland (Karjala, n = 129), as well as Finnish domestic populations (*R. t. tarandus*) from northern Ostrobothnia (Kuusamo and Pudasjärvi localities; Pohjois-Pohjanmaa, n = 28), southern Lapland (Rovaniemi, Savukoski and Sodankylä localities; Etelä-Lappi, n = 49) and northern Lapland (Enontekiö and Inari localities; Pohjois-Lappi, n = 43). Finnish domestic reindeer (i.e. Pohjois-Pohjanmaa, Etelä-Lappi and Pohjois-Lappi) and wild Finnish forest reindeer (i.e. Länsi-Suomi and Karjala) are from the collection of the Biodiversity Unit of the University of Oulu, and wild Norwegian mountain reindeer (i.e. Hardangervidda and Knutshø) currently housed at the ArScAn laboratory (UMR 7041). The sample included 581 teeth (327 m1 and 254 m2). The imbalance in the numbers of the two molars is due to material preservation (i.e. broken teeth were not taken into account) and the difference in their eruption stage. All the teeth were embedded in their mandibles. The details of samples used for each tooth are given in Table 1. Finally, sex was known for 353 individuals and age for 235.

2. Archaeological sites

The archaeological reindeer teeth analysed in this study came from two Sámi dwelling sites and two Sámi marketplaces in present-day Finland (Figure 1). The sites date from ca. AD 1,300 to 1,800 [1, 2]. Ninety reindeer molars (36 m1 and 54 m2) were included in the geometric morphometrics analyses (Table 1).

Juikenttä: Juikenttä is located in the forest region of Lapland in Sodankylä. According to the material culture typology, the site was occupied by Sámi from the Middle Ages to Early Modern Times ca. AD 1050-1650 [3]. Fishing and hunting wild feathered game played an important role in the livelihood of the villages of Sodankylä. Historically, reindeer herding was not practised in this region during the 11th and 12th centuries. However, the discovery of pieces of reindeer harness at Juikenttä suggests that reindeer were used as draught animals at some point [3]. The faunal assemblage was dominated by reindeer [1, 2]. The reindeer remains are derived from all body parts, suggesting that the animals were slaughtered locally with extensive breakage of long bones to enable marrow extraction [1]. Recent radiocarbon dating performed directly on the reindeer bones has aged them to AD 1292-1662 [2].

Nukkumajoki: Nukkumajoki is located in Inari, near the boreal forest tree line. The site is part of a series of large winter villages occupied by the Sámi from the 15th century to the mid-16th century [4-6]. More precisely, the Nukkumajoki 2 site is dated to ca. 1480-1580 [1]. The majority of bone fragments are from reindeer [1, 2]. Some pieces of reindeer harness were discovered at the site, suggesting that the reindeer were not only hunted but also used as draught animals [5]. The skeletal representation also shows that the reindeer were slaughtered on site [1]. Recent radiocarbon dating performed directly on the reindeer bones has provided an age to AD 1447-modern, with most samples dating from the 16th and 17th centuries [2].

Markkina: Markkina is also near the boreal forest tree line in Enontekiö. It was an annual marketplace established in 1604 and abandoned in 1826 [7]. The site has been dated from various artefacts including coins. Markkina was the centre of three local Sámi communities (*siida*): Rounala, which had already

practised reindeer herding at the end of the 16th century; Suonttavaara which had started reindeer herding at the beginning of the 17th century; and Peltojärvi which had started herding in the mid-18th century [8]. The bone assemblage was dominated by reindeer and an analysis of reindeer skeletal frequencies suggests that all body parts were present, meaning the animals were slaughtered locally [1].

Pappila: Pappila is located in Utsjoki in the fell region. According to the coins, this marketplace was probably mainly occupied between 1640 and 1820 [7]. Historically, most of the Utsjoki *siida* practised reindeer herding in the 17th century, but subsistence was mainly based on hunting and fishing [7]. At Pappila, reindeer were also by far the most represented animal in the faunal assemblage and their skeletal profile also suggests local slaughter of animals [1].

3. Age and establishment of wear classes

Four wear classes were defined to test the impact of wear on the overall morphology of the teeth.

After several attempts to obtain adequate and reproducible criteria to assess wear on *Rangifer* teeth, we selected four variables, inspired by the work of Brown and Chapman [9] and Dudley Furniss-Roe [10] on scoring schemes applied to red deer teeth: **A**) exposed dentine connections (with wear, more dentine is exposed so that a continuous "link" is seen between the cusps); **B**) the loss of folds on mesiolingual cusps; **C**) disappearance of the infundibulum (worn away); **D**) loss of enamel contact points between the teeth (Supplementary Figure S1).

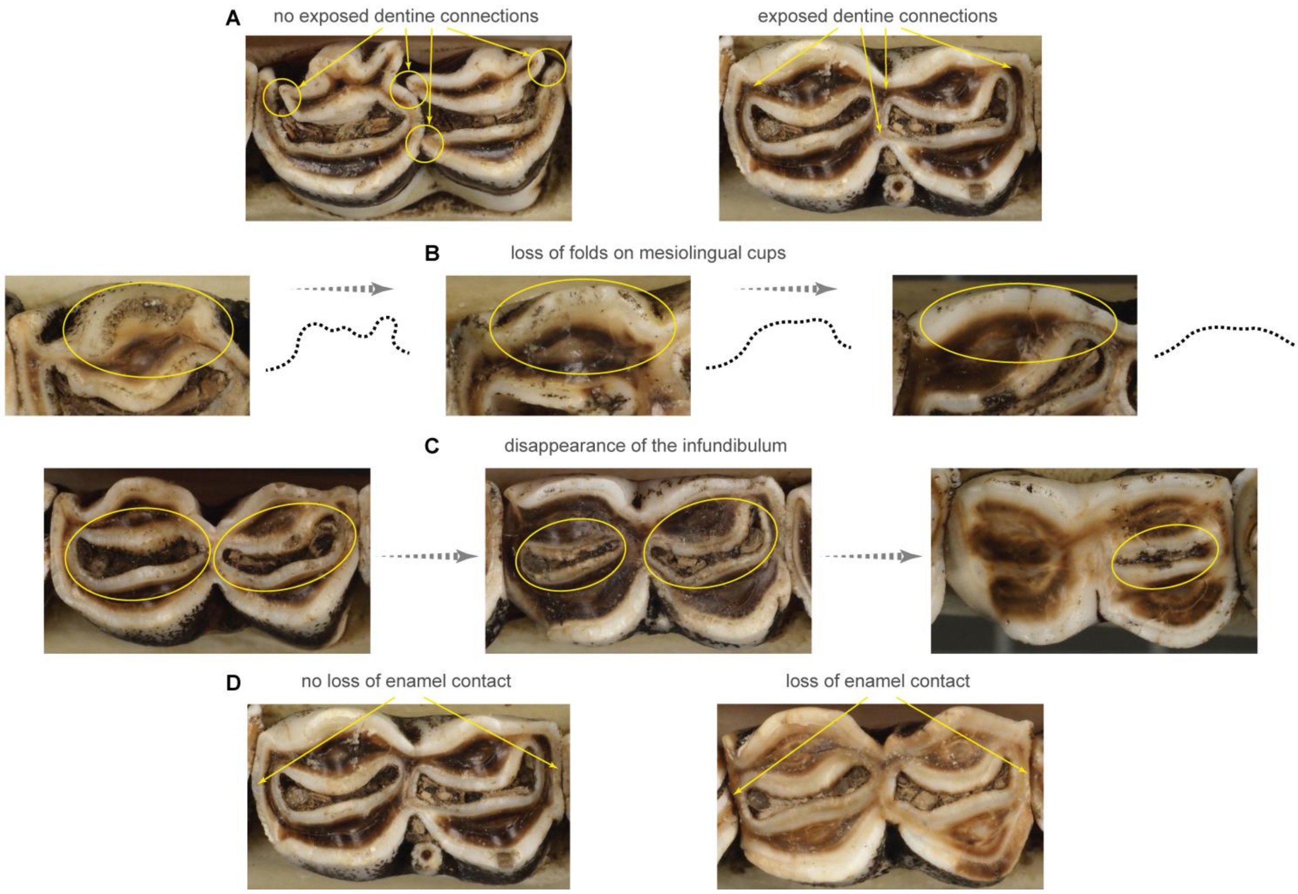

Supplementary Figure S1. Tooth criteria used for the definition of wear classes.

The wear classes were classified into the following categories (Supplementary Figure S2):

1) **Class 0**: Unworn or very lightly worn cusps with no exposed dentine connections between the mesial and distal lobes;

2) **Class 1**: minimally worn cusps with exposed dentine connections between the mesial and distal lobes, with still folded lingual cusps, and without or only moderate loss of enamel contacts;

3) **Class 2**: more advanced wear with exposed dentine connections, an attenuation of the folds on the mesiolingual lingual cusps, and moderate loss of enamel contacts;

4) **Class 3**: well advanced wear with exposed dentine connections, no further folding of the lingual cusps and loss of enamel contacts. The wear progressively reaches the bottom of the infundibulum;

5) **Class 4**: very advanced wear with disappearance of the infundibulums.

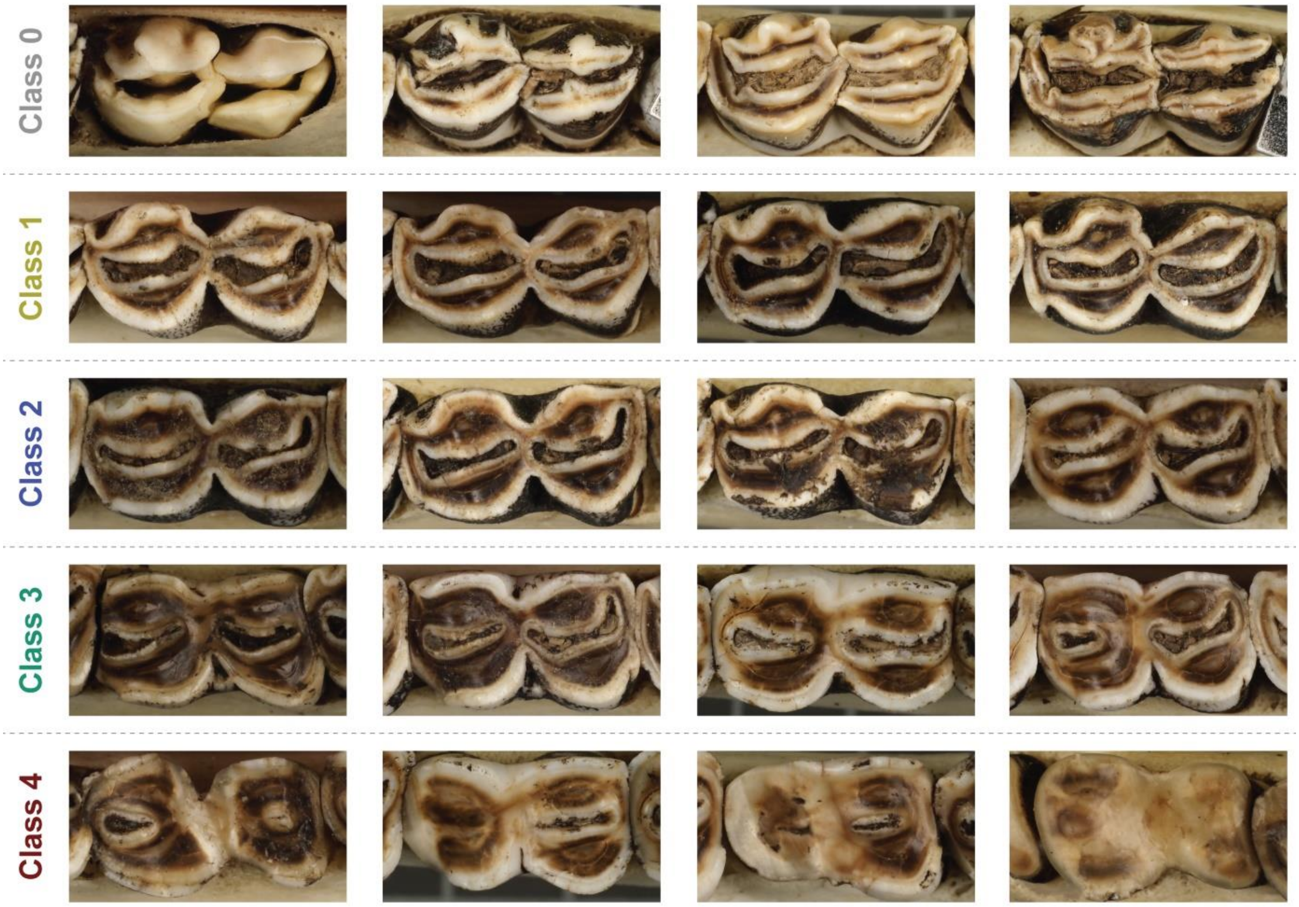

Supplementary Figure S2. Progress of tooth wear observed in modern reindeer.

4. Tooth landmarks

For each m1 and m2, the occlusal view was photographed using a standardised protocol: first, the vestibular cusps were levelled so that the occlusal surface was parallel to the focal plane and then the position of the tooth was adjusted in the vestibulo-lingual plane so that the vestibular and lingual sides of the crown were either no longer visible (in the case of fairly worn teeth) or equally visible on both sides (in the case of less worn teeth). Only left teeth were selected for digitisation to avoid symmetric redundancy, but when no left teeth were available, right teeth were selected and mirrored before analysis. There is currently no set of landmarks for the geometric morphometric study of reindeer teeth published in the academic canon. Among the recent studies that have investigated ungulate tooth morphology, there would appear to be no consensus on methodological approaches to the positioning of landmarks. For example in caprines, landmarks have been positioned along the outer outline of the tooth [11]. In equids, landmarks were placed in the centre of the enamel thickness [12] while in bovids they were positioned at the junction between dentine and enamel [13].

We also aimed to apply the same protocol on both m1 and m2, on a maximum of individuals of different ages (including all wear classes, if possible), as well as on archaeological specimens that may present different states of preservation (i.e. taphonomy). Thus, to properly define the position of our landmarks and their relevance, we first quantified the number of individuals showing loss of enamel contact (see **a** and **h** in Supplementary Figure S3) and exposed dentine connections (see **b** to **g** in Supplementary Figure S3).

This preliminary work was conducted on an even larger sample of individuals (508 individuals; 1,000 teeth), which included individuals from other localities or individuals not usable in geometric morphometric studies (i.e. teeth too worn and/or from unknown origin, age and sex). Regarding enamel, we observed that the more worn the tooth, the more individuals showed loss of enamel contact on the mesial and distal edges of the tooth. For the same wear stage, m1 is more impacted than m2, and the mesial edge (contact between p4/m1 or m1/m2) is generally more impacted than the distal edge (contact between m1/m2 or m2/m3). Thus, the parastylid tends to disappear with wear, unlike the entostylid. The individuals least affected by this mesial and distal loss of enamel were those individuals from Class 0. From Class 2 onwards, the majority of individuals showed a loss of enamel contact, and from Class 3 onwards, this affected almost all individuals. Regarding the exposed dentine connections, this concerns more than half of the m1 in individuals from Class 0 to 1 and a large proportion of m2 in individuals in Class 1. From Class 2 onwards, almost all individuals exposed dentine connections for both m1 and m2.

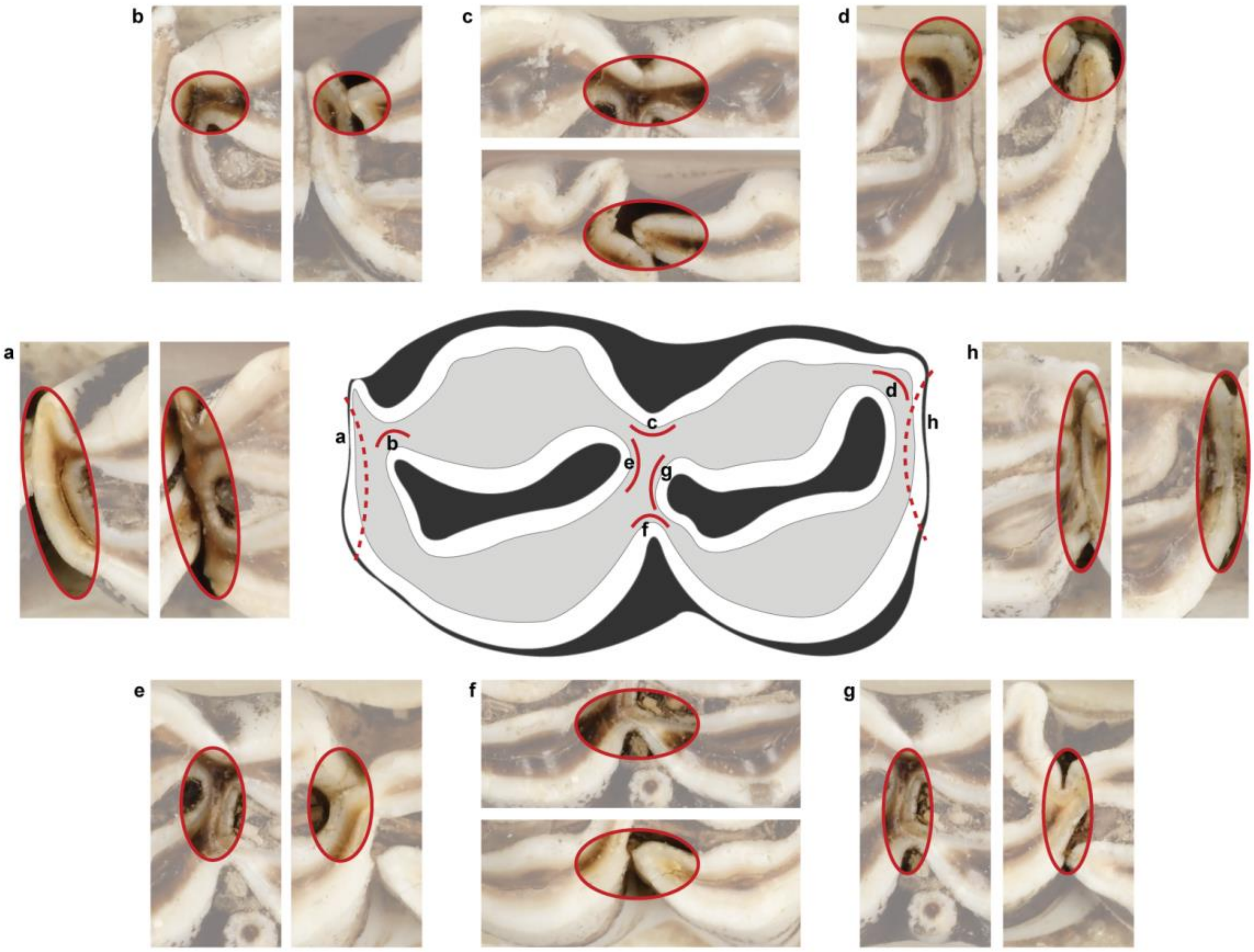

Supplementary Figure S3. Morphological criteria examined prior to landmark selection. **a**: LEC between p4/m1 or m1/m2; **b**: EDC between the lingual and vestibular sides; **c**: EDC between the mesial and distal sides; **d**: EDC between the lingual and vestibular sides; **e**: EDC between the lingual and vestibular sides; **f**: EDC between the mesial and distal sides; **g**: EDC between the lingual and vestibular sides; **h**: LEC between m1/m2 or m2/m3. LEC: loss of enamel contact; EDC: exposed dentine connections

With tooth wear, a majority of individuals had loss of enamel in the mesial or distal part of the tooth, which prevented landmarks from being placed along the outer outline of the tooth. This is because the loss of dental material in these areas makes it impossible to identify the same homologous points throughout the different wear classes. Thus ,the mesial (i.e. between the parastylid and the protoconid) and distal parts (i.e. between the entostylid and the hypoconid) were excluded from the landmarks. In addition, most individuals exposed dentine connections in occlusal view, indicating that the enamel-dentine junction is visible in most individuals (except for very young individuals in Class 0, a few young individuals in Class 1 and a few very old individuals in Class 4). This offers the potential to place landmarks along most of the tooth on the inner edge of the enamel.

Less affected by tooth wear than the outer enamel surface, the enamel-dentine junction is more conservative and maintains a more ancestral morphology than the outer enamel surface [14, 15]. Furthermore, numerous studies on the enamel-dentine junction have shown that its morphology contains information on taxonomy and phylogenetic relationships [16-20]. Based on macroevolutionary approaches, these studies have shown

that the anatomical variations are well expressed at the enamel-dentine junction, and thus successfully discriminate between species or differentiate between molars. Finally, the enamel-dentine junction is clearly distinguishable in the archaeological specimens, enabling the positioning of landmarks. Furthermore, depending on the taphonomy of the archaeological assemblages, many teeth showed breaks in the enamel, but the outer part of the enamel-dentine junction (i.e. on the occlusal surface) was very often intact.

Thus, the shape of the m1 and the m2 was estimated using a new 2D protocol involving nine landmarks (LM) positioned on the inner edge of the enamel, i.e. at the enamel-dentine junction, to limit biases related to external enamel wear (Figure 6). These landmarks correspond to the points of maximum curvature, which makes it difficult to quantify the remaining tooth shape using traditional landmarks. Thus, semilandmarks have been included on curves to help capture the tooth conformation of the enamel-dentine junction. A total of 66 equidistant sliding semilandmarks (SSLM) were distributed along seven curves: seven SSLM between LM1 and LM2, eight SSLM between LM2 and LM3, seven SSLM between LM3 and LM4, 11 SSLM between LM4 and LM5, nine SSLM between LM5 and LM6, 14 SSLM between LM7 and LM8 and 10 SSLM between LM8 and LM9. The landmark and semi-landmark coordinates were acquired by a single operator (MP) from digital photographs using tpsDig2 v.2.16 [21].

5. Results

5.1. Anatomical distinction of lower molars and wear impact

The factorial MANOVA found significant differences in shape among the two lower molar and the four wear classes investigated (Supplementary Table S1). Although the wear signal was stronger than the anatomical signal, with 47.5% against 10.2% of shape variation explained, respectively, significant interaction between both factors was found to explain 1.3% of the overall variation. This indicates that the wear varies slightly across the two teeth and therefore a separate investigation of the taxonomic resolution for each tooth is required.

	Df	Sum Sq	Mean Sq	Rsq	*F*-value	*P*-value
Tooth	1	0.058	0.058	0.102	114.966	**< 2.2e-16**
Class	3	0.211	0.070	0.475	140.031	**< 2.2e-16**
Tooth : Class	2	0.007	0.004	0.013	7.045	**9.49e-04**

Supplementary Table S1. Results of the factorial MANOVA which tested the differences between the two lower molar (Tooth) and the four wear class (Class) of our sample, as well as the interaction between the two factors (Tooth : Class).

In our sample, the factorial analysis of variance found significant differences in size and shape in both molars ($P < 0.05$). When all wear classes were involved, m1 has a significantly smaller centroid size than m2 (Supplementary Figure S5A). The discriminant model found correct molar identification for more than 90% of the classifications after cross-validation. This shows that this model can accurately distinguish an isolated lower molar from the archaeological record, regardless of the stage of wear. The main morphological differences between m1 and m2 are described in the Supplementary Text 5.1 and. Supplementary Figure S5B. The main morphological differences between m1 and m2 are that the vestibulo-lingual diameter of the mesial lobe is smaller than the vestibulo-lingual diameter of the distal lobe on m1, while on m2, the opposite is true (Supplementary Figure S5B), as previously described by some palaeontologists [22].

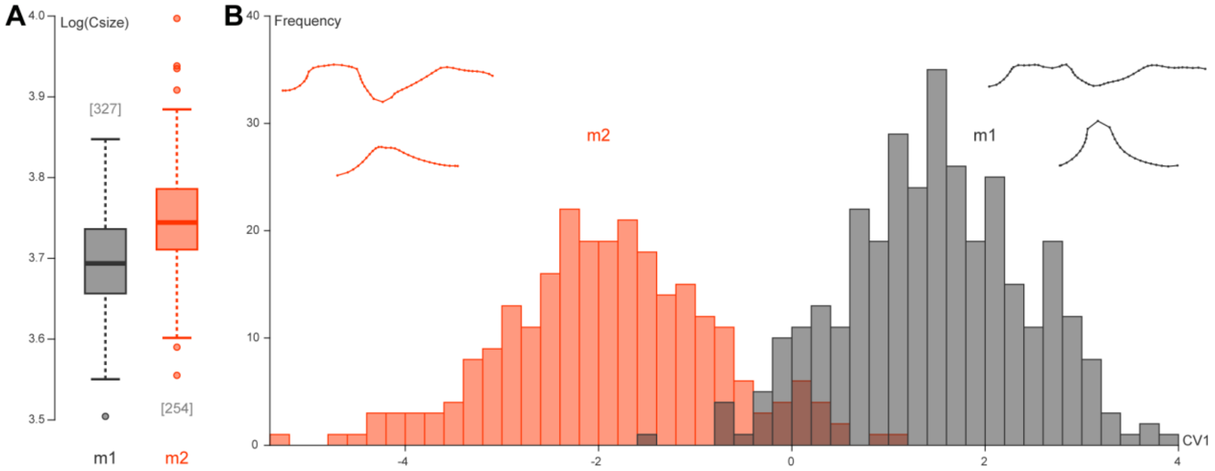

Supplementary Figure S4. A: Boxplots of the variation in log-transformed centroid size (Csize) according to the tooth (m1 and m2). **B**: Canonical analysis of variance (CVA) comparing the tooth shape (m1/m2) with the visualisation of shape differences along axis 1 (CV1) at the extreme values. The numbers in square brackets represent the number of teeth analysed in each group.

**m1 - Size**			**m2 - Size**		
	Class 1	Class 2		Class 1	Class 2
Class 2	**8.123e-04**	-	Class 2	0.312	-
Class 3	**2.039e-11**	**2.043e-05**	Class 3	**7.300e-03**	**1.226e-06**
**m1 - Shape**			**m2 - Shape**		
	Class 1	Class 2		Class 1	Class 2
Class 2	**< 2.2e-16**	-	Class 2	0.209	-
Class 3	**< 2.2e-16**	**< 2.2e-16**	Class 3	**2.099e-04**	**< 2.2e-16**

Supplementary Table S2. Multi-test comparisons (*P*-values) of size and shape data between the different wear classes for m1 and m2 (pairwise Wilcoxon rank tests after the ‘Benjamini-Hochberg’ correction). A significant contribution was considered for a *P*-value < 0.05 (**in bold**).

Individuals in Class 1 showed a mesiolingual fold between the metaconid and the parastyle and a lingual cusp fold between the metaconid and the metastylid both well-expressed (Supplementary Figure S6C). In contrast to Class 3, these folds were attenuated while the interlobal grooves were deeper, especially on the vestibular side. To summarise, on both teeth, the enamel folds on the lingual side diminished with wear, and the interlobal groove on the vestibular side became deeper

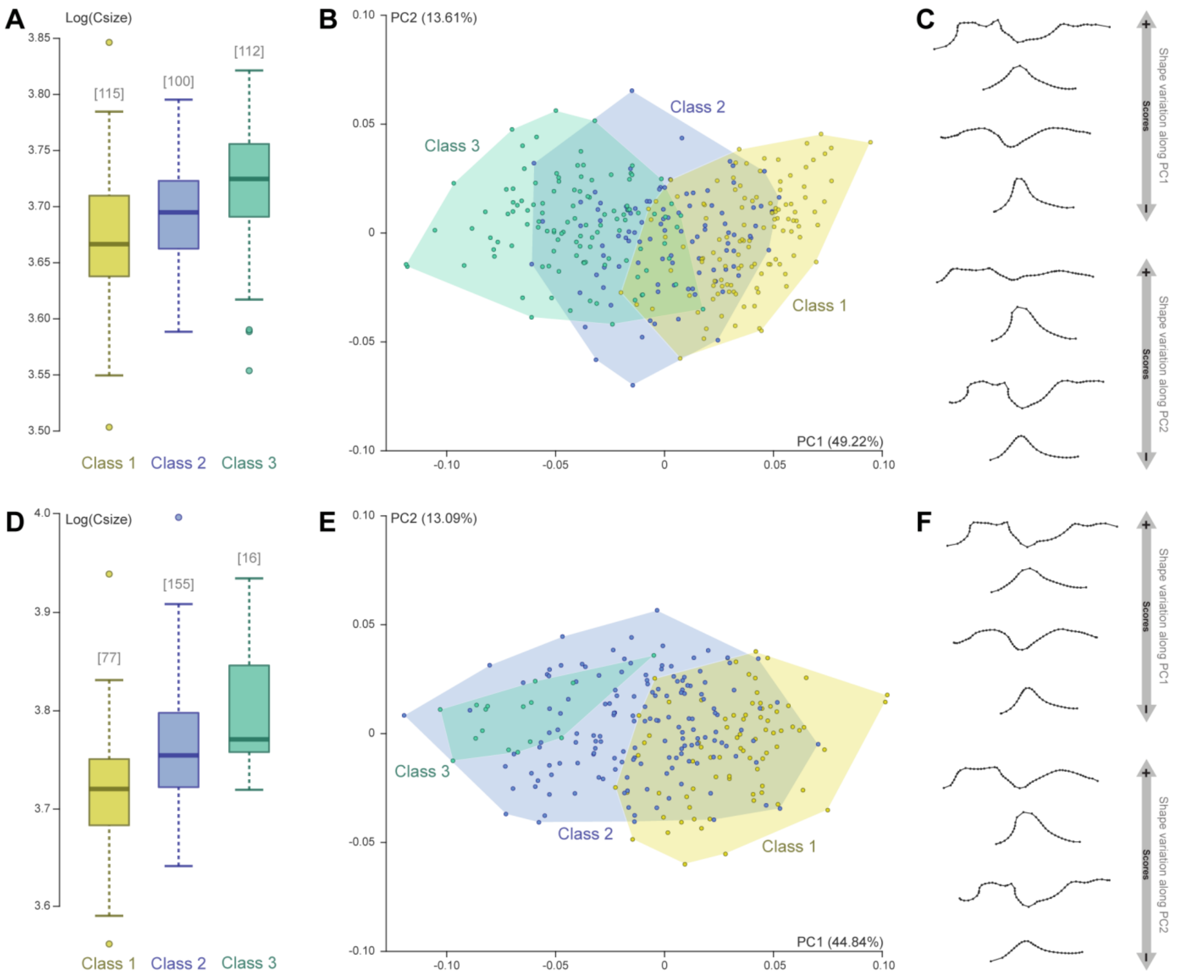

Supplementary Figure S5. Size and shape analysis of m1 (**A–C**) and m2 (**D–F**) in modern populations. Boxplots of the variation in log-transformed centroid size (Csize) according to the tooth wear class for m1 (**A**) and m2 (**D**). The numbers in square brackets represent the number of teeth analysed in each category. Scatter plots of the two first axes (PC1 and PC2) of the principal component analyses performed on the shape data according to the tooth wear class for m1 (**B**) and m2 (**E**). The proportion of the total variance expressed by the PC1 and PC2 axes, respectively, is indicated in brackets. Visualisation of shape variation via deformation of the mean shape along the negative and positive PC1 and PC2 values for m1 (**C**) and m2 (**F**).

**m1**	Df	Sum Sq	Mean Sq	Rsq	*F*-value	*P*-value
Class	2	0.207	0.103	0.553	253.526	**< 2.2e-16**
Population	6	0.012	0.002	0.034	4.928	**7.87e-05**
Sex	2	0.004	0.002	0.011	4.595	**0.011**
Class : Population	12	0.007	0.001	0.020	0.001	0.135
Class : Sex	4	0.001	0.001	0.001	0.289	0.885
**m2**	Df	Sum Sq	Mean Sq	Rsq	*F*-value	*P*-value
Class	3	0.069	0.023	0.334	44.090	**< 2.2e-16**
Population	6	0.011	0.002	0.055	3.631	**0.001**
Sex	2	0.001	0.001	0.002	0.456	0.634
Class : Population	12	0.006	0.001	0.029	0.975	0.474
Class : Sex	5	0.004	0.001	0.020	1.490	0.194

Supplementary Table S3. Results of the factorial MANOVA which tested the differences between the wear class (Class), the seven populations (Population) and sex (Sex) of our sample, as well as the interaction between the two factors (Class : Population and Class : Sex) for each tooth.

5.2. Molars size and shape variation among modern Fennoscandian reindeer populations

	Df	Sum Sq	Mean Sq	*F*-value	*P*-value
**m1 - Class 1**					
Subspecies (ssp.)	1	0.003	0.003	0.97	0.33
Status	2	0.033	0.017	5.08	**7.69e-03**
Sex	2	0.008	0.004	1.15	0.32
Populations	6	0.056	0.009	2.90	**0.01**
ssp. × sex	5	0.024	0.005	1.37	0.24
status × sex	8	0.086	0.011	3.59	**1.01e-03**
populations × sex	17	0.114	0.007	2.27	**6.38e-03**
**m1 - Class 2**					
Subspecies (ssp.)	1	0.0203	0.0203	10.88	**1.35e-03**
Status	2	0.0352	0.0176	10.14	**1.00e-04**
Sex	2	0.0003	0.0002	0.08	0.93
Populations	6	0.0506	0.0084	5.131	**1.36e-04**
ssp. × sex	5	0.0263	0.0053	2.79	0.02
status × sex	7	0.0378	0.0054	2.99	**7.08e-03**
populations × sex	17	0.0619	0.0036	2.11	0.01
**m1 - Class 3**					
Subspecies (ssp.)	1	0.054	0.054	24.12	**3.17e-06**
Status	2	0.065	0.032	14.84	**1.99e-06**
Sex	2	0.023	0.011	4.47	**0.01**
Populations	6	0.078	0.013	6.04	**1.86e-05**
ssp. × sex	5	0.072	0.014	6.61	**2.16e-05**
status × sex	7	0.078	0.011	5.16	**4.53e-05**
populations × sex	18	0.105	0.006	2.72	**9.25e-04**
**m2 - Class 1**					
Subspecies (ssp.)	1	0.021	0.021	6.59	**0.01**
Status	2	0.021	0.011	3.37	**0.04**
Sex	2	0.002	0.001	0.29	0.75
Populations	6	0.054	0.009	3.10	**9.45e-03**
ssp. × sex	5	0.028	0.006	1.74	0.14
status × sex	7	0.048	0.007	2.26	**0.04**
populations × sex	16	0.086	0.005	1.88	**0.04**
**m2 - Class 2**					
Subspecies (ssp.)	1	0.105	0.105	42.46	**9.85e-10**
Status	2	0.112	0.056	22.79	**2.20e-09**
Sex	2	0.053	0.026	9.24	**1.63e-04**
Populations	6	0.150	0.025	11.11	**3.28e-10**
ssp. × sex	5	0.137	0.027	11.77	**1.33e-09**
status × sex	8	0.147	0.018	7.96	**7.19e-09**
populations × sex	19	0.184	0.010	4.35	**1.78e-07**

Supplementary Table S4. ANOVA results for each tooth and wear class analysed according to the different categories. A significant contribution was considered for *P*-value < 0.05 (**in bold**).

The results of the ANOVA on size data reveal significant differences between almost all categories for all wear classes of both teeth ($P < 0.05$; Supplementary Table S2). However, there was no significant difference in size between the subspecies in Class 1 of the m1, between the sexes in Classes 1 and 2 of m1 and in Class 1 of m2, between 'subspecies + sex' in Classes 1 and 2 of m1 and in Class 1 of m2, and between 'populations + sex' in Class 2 of m1.

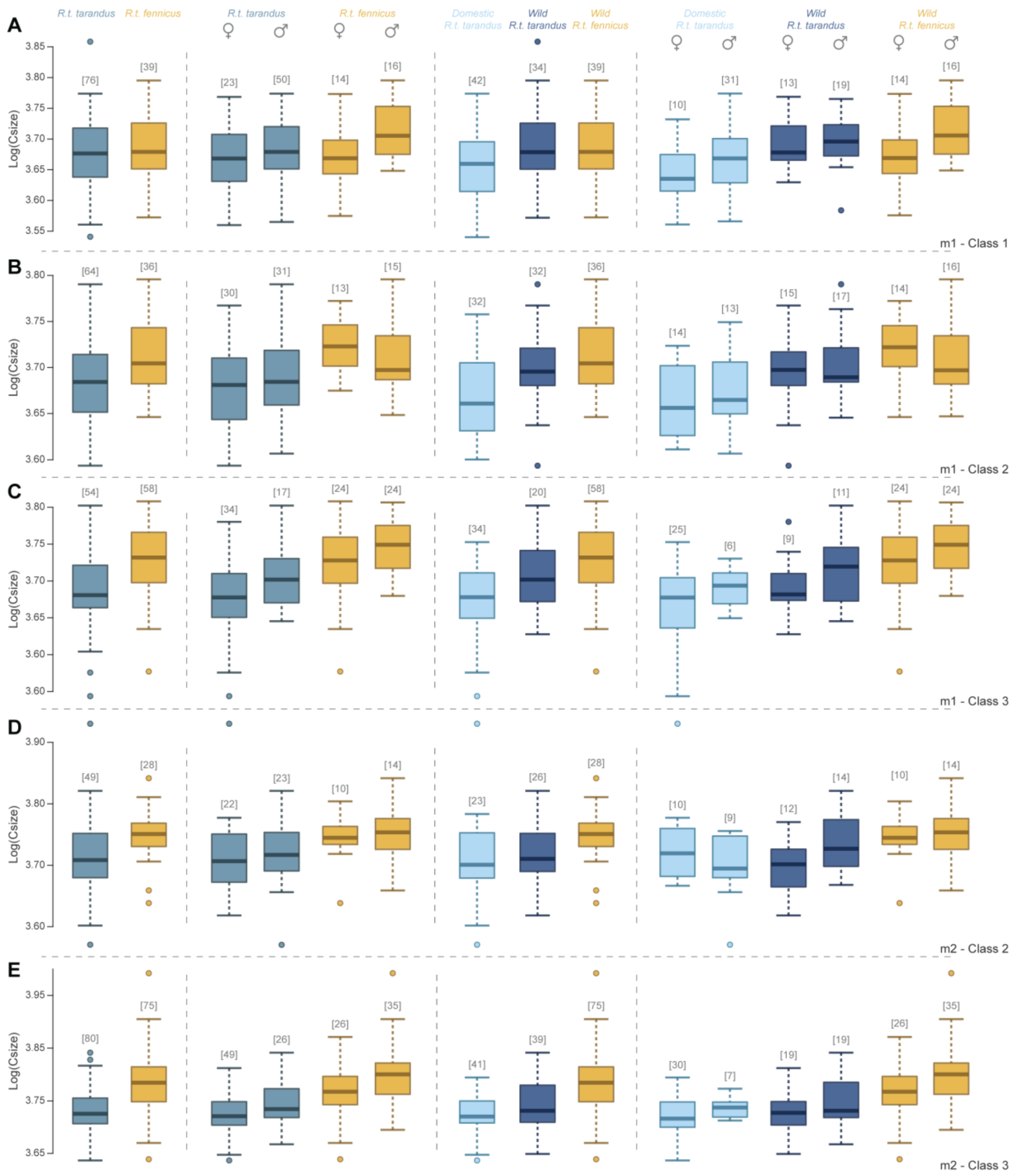

Supplementary Figure S6. Boxplots of the variation in log-transformed centroid size (Csize) according to the subspecies (*R.t. tarandus* and *R.t. fennicus*), sex (female = ♀ and male = ♂) and status (domestic reindeer, wild mountain reindeer and wild forest reindeer) for m1-Class1 (**A**), m1-Class2 (**B**), m1-Class3 (**C**), m2-Class1 (**D**) and m2-Class2 (**E**). The numbers in square brackets represent the number of teeth analysed in each category.

Wilcoxon test	m1 – Class 1	m1 – Class 2	m1 – Class 3	m2 – Class 1	m2 – Class 2
			R. t. fennicus		
R. t. tarandus	0.380	**2.984e-03**	**2.365e-06**	**2.246e-03**	**2.213e-09**

Supplementary Table S5. Multi-test comparisons (*P*-values) of log-transformed centroid sizes for each wear class for m1 and m2 between the different reindeer subspecies. A significant contribution was considered for *P*-value < 0.05 (**in bold**).

**m1 – Class 1**		
	Wild *R. t. fennicus*	Wild *R. t. tarandus*
Wild *R. t. tarandus*	0.423	-
Domestic *R. t. tarandus*	0.056	**0.019**
**m1 – Class 2**		
	Wild *R. t. fennicus*	Wild *R. t. tarandus*
Wild *R. t. tarandus*	0.167	-
Domestic *R. t. tarandus*	**6.700e-04**	**9.050e-03**
**m1 – Class 3**		
	Wild *R. t. fennicus*	Wild *R. t. tarandus*
Wild *R. t. tarandus*	**0.031**	-
Domestic *R. t. tarandus*	**2.676e-06**	0.087
**m2 – Class 1**		
	Wild *R. t. fennicus*	Wild *R. t. tarandus*
Wild *R. t. tarandus*	0.019	-
Domestic *R. t. tarandus*	0.019	0.531
**m2 – Class 2**		
	Wild *R. t. fennicus*	Wild *R. t. tarandus*
Wild *R. t. tarandus*	**1.797e-04**	-
Domestic *R. t. tarandus*	**9.342e-09**	0.129

Supplementary Table S6. Multi-test comparisons (*P*-values) of log-transformed centroid sizes for each wear class for m1 and m2 between the different reindeer status (i.e. domestic reindeer, wild mountain reindeer and wild forest reindeer) (pairwise Wilcoxon rank tests after the 'Benjamini-Hochberg' correction). A significant contribution was considered for *P*-value < 0.05 (**in bold**).

**m1 – Class 1**			
	R. t. fennicus ♀	*R. t. fennicus* ♂	*R. t. tarandus* ♀
R. t. fennicus ♂	0.107	-	-
R. t. tarandus ♀	0.963	0.095	-
R. t. tarandus ♂	0.588	0.109	0.588
**m1 – Class 2**			
	R. t. fennicus ♀	*R. t. fennicus* ♂	*R. t. tarandus* ♀
R. t. fennicus ♂	0.400	-	-
R. t. tarandus ♀	**0.014**	0.089	-
R. t. tarandus ♂	**0.026**	0.144	0.432
**m1 – Class 3**			
	R. t. fennicus ♀	*R. t. fennicus* ♂	*R. t. tarandus* ♀
R. t. fennicus ♂	0.298	-	-
R. t. tarandus ♀	**1.100e-03**	**9.200e-06**	-
R. t. tarandus ♂	0.162	**0.014**	0.162
**m2 – Class 1**			
	R. t. fennicus ♀	*R. t. fennicus* ♂	*R. t. tarandus* ♀
R. t. fennicus ♂	0.752	-	-
R. t. tarandus ♀	0.184	0.136	-
R. t. tarandus ♂	0.269	0.172	0.608
**m2 – Class 2**			
	R. t. fennicus ♀	*R. t. fennicus* ♂	*R. t. tarandus* ♀
R. t. fennicus ♂	**0.038**	-	-
R. t. tarandus ♀	**3.632e-04**	**2.363e-09**	-
R. t. tarandus ♂	0.058	**1.019e-04**	0.083

Supplementary Table S7. Multi-test comparisons (*P*-values) of log-transformed centroid sizes for each wear class for m1 and m2 between the different reindeer 'subspecies + sex' (pairwise Wilcoxon rank tests after the 'Benjamini-Hochberg' correction). A significant contribution was considered for *P*-value < 0.05 (**in bold**).

**m1 – Class 1**	*Wild ♀ R. t. fennicus*	*Wild ♂ R. t. fennicus*	*Wild ♀ R. t. tarandus*	*Wild ♂ R. t. tarandus*	*Domestic ♀ R. t. tarandus*
Wild ♂ R. t. fennicus	0.107	-	-	-	-
Wild ♀ R. t. tarandus	0.400	0.365	-	-	-
Wild ♂ R. t. tarandus	0.179	0.526	0.633	-	-
Domestic ♀ R. t. tarandus	0.382	0.061	0.107	0.071	-
Domestic ♂ R. t. tarandus	0.912	0.089	0.382	0.172	0.365

**m1 – Class 2**	*Wild ♀ R. t. fennicus*	*Wild ♂ R. t. fennicus*	*Wild ♀ R. t. tarandus*	*Wild ♂ R. t. tarandus*	*Domestic ♀ R. t. tarandus*
Wild ♂ R. t. fennicus	0.569	-	-	-	-
Wild ♀ R. t. tarandus	0.174	0.756	-	-	-
Wild ♂ R. t. tarandus	0.306	0.756	0.850	-	-
Domestic ♀ R. t. tarandus	0.053	0.063	0.165	0.063	-
Domestic ♂ R. t. tarandus	0.063	0.165	0.320	0.134	0.756

**m1 – Class 3**	*Wild ♀ R. t. fennicus*	*Wild ♂ R. t. fennicus*	*Wild ♀ R. t. tarandus*	*Wild ♂ R. t. tarandus*	*Domestic ♀ R. t. tarandus*
Wild ♂ R. t. fennicus	0.447	-	-	-	-
Wild ♀ R. t. tarandus	0.156	**0.025**	-	-	-
Wild ♂ R. t. tarandus	0.472	0.156	0.529	-	-
Domestic ♀ R. t. tarandus	**2.500e-03**	**4.100e-05**	0.447	0.156	-
Domestic ♂ R. t. tarandus	0.172	**0.033**	0.953	0.463	0.472

**m2 – Class 1**	*Wild ♀ R. t. fennicus*	*Wild ♂ R. t. fennicus*	*Wild ♀ R. t. tarandus*	*Wild ♂ R. t. tarandus*	*Domestic ♀ R. t. tarandus*
Wild ♂ R. t. fennicus	0.806	-	-	-	-
Wild ♀ R. t. tarandus	0.196	0.137	-	-	-
Wild ♂ R. t. tarandus	0.635	0.428	0.196	-	-
Domestic ♀ R. t. tarandus	0.594	0.428	0.428	0.769	-
Domestic ♂ R. t. tarandus	0.196	0.143	0.972	0.311	0.428

**m2 – Class 2**	*Wild ♀ R. t. fennicus*	*Wild ♂ R. t. fennicus*	*Wild ♀ R. t. tarandus*	*Wild ♂ R. t. tarandus*	*Domestic ♀ R. t. tarandus*
Wild ♂ R. t. fennicus	0.054	-	-	-	-
Wild ♀ R. t. tarandus	**0.044**	**2.554e-04**	-	-	-
Wild ♂ R. t. tarandus	0.200	**2.086e-03**	0.522	-	-
Domestic ♀ R. t. tarandus	**6.875e-04**	**1.950e-08**	0.469	0.121	-
Domestic ♂ R. t. tarandus	0.121	**4.962e-03**	0.778	0.778	0.235

Supplementary Table S8. Multi-test comparisons (*P*-values) of log-transformed centroid sizes for each wear class for m1 and m2 between the different reindeer 'status + sex' (pairwise Wilcoxon rank tests after the 'Benjamini-Hochberg' correction). A significant contribution was considered for *P*-value < 0.05 (**in bold**).

	Df	Pillai	Approx F	Num Df	Den Df	Pr	CCV (%)
**m1 - Class 1**							
Subspecies (ssp.)	1	0.595	7.346	19	95	**1.034e-11**	84.35
Status	2	1.026	5.271	38	190	**4.969e-15**	67.83
Sex	2	0.407	1.276	38	190	0.147	56.52
Populations	6	2.051	2.597	114	570	**1.689e-13**	44.35
**m1 - Class 2**							
Subspecies (ssp.)	1	0.610	8.116	16	83	**2.963e-11**	91.00
Status	2	1.017	5.368	32	166	**2.147e-13**	73.00
Sex	2	0.391	1.262	32	166	0.176	48.00
Populations	6	1.791	2.207	96	498	**1.967e-08**	47.00
**m1 - Class 3**							
Subspecies (ssp.)	1	0.595	10.165	14	97	**1.256e-13**	85.71
Status	2	0.756	4.207	28	194	**1.107e-09**	68.75
Sex	2	0.422	1.854	28	194	**8.373e-03**	58.04
Populations	6	1.270	1.860	84	582	**8.373e-03**	48.21
**m2 - Class 1**							
Subspecies (ssp.)	1	0.566	4.517	17	59	**7.24e-06**	77.92
Status	2	0.896	2.817	34	118	**1.959e-05**	57.14
Sex	2	0.535	1.267	34	118	0.177	45.45
Populations	6	2.241	2.069	102	354	**5.285e-07**	48.05
**m2 - Class 2**							
Subspecies (ssp.)	1	0.540	10.130	16	138	**< 2.2e-16**	80.00
Status	2	0.813	5.906	32	276	**< 2.2e-16**	72.26
Sex	2	0.326	1.680	32	276	**0.015**	57.42
Populations	6	1.298	2.381	96	828	**7.384e-11**	50.32

Supplementary Table S9. Results of the MANOVA tests for each element analysed and the percentage of correct cross-validated classification (CCV) according to the different categories. A significant contribution was considered for *P*-value < 0.05 (**in bold**)

Wilcoxon test	**m1 – Class 1**	**m1 – Class 2**	**m1 – Class 3**	**m2 – Class 1**	**m2 – Class 2**
			R. t. fennicus		
R. t. tarandus	**3.931e-09**	**0.011**	0.664	**5.056e-06**	**9.075e-06**

Supplementary Table S10. Multi-test comparisons (*P*-values) on shape data for each wear class for m1 and m2 between the different reindeer subspecies. A significant contribution was considered for *P*-value < 0.05 (**in bold**).

	Wild *R. t. fennicus*	Wild *R. t. tarandus*
**m1 – Class 1**		
Wild *R. t. tarandus*	**9.812e-07**	-
Domestic *R. t. tarandus*	**3.723e-08**	0.823
**m1 – Class 2**		
	Wild *R. t. fennicus*	Wild *R. t. tarandus*
Wild *R. t. tarandus*	0.869	-
Domestic *R. t. tarandus*	**6.543e-05**	**9.454e-05**
**m1 – Class 3**		
	Wild *R. t. fennicus*	Wild *R. t. tarandus*
Wild *R. t. tarandus*	0.186	-
Domestic *R. t. tarandus*	0.6303	0.186
**m2 – Class 1**		
	Wild *R. t. fennicus*	Wild *R. t. tarandus*
Wild *R. t. tarandus*	**1.012e-03**	-
Domestic *R. t. tarandus*	**2.871e-05**	0.178
**m2 – Class 2**		
	Wild *R. t. fennicus*	Wild *R. t. tarandus*
Wild *R. t. tarandus*	**0.011**	-
Domestic *R. t. tarandus*	**1.602e-05**	0.058

Supplementary Table S11. Multi-test comparisons (*P*-values) on shape data for each wear class for m1 and m2 between the different reindeer status (i.e. domestic reindeer, wild mountain reindeer and wild forest reindeer) (pairwise Wilcoxon rank tests after the 'Benjamini-Hochberg' correction). A significant contribution was considered for *P*-value < 0.05 (**in bold**).

**m1 – Class 1**			
	R. t. fennicus ♀	*R. t. fennicus* ♂	*R. t. tarandus* ♀
R. t. fennicus ♂	0.377	-	-
R. t. tarandus ♀	**3.837e-05**	**8.682e-06**	-
R. t. tarandus ♂	**1.757e-04**	**1.979e-04**	0.377
**m1 – Class 2**			
	R. t. fennicus ♀	*R. t. fennicus* ♂	*R. t. tarandus* ♀
R. t. fennicus ♂	0.650	-	-
R. t. tarandus ♀	0.059	0.035	-
R. t. tarandus ♂	0.059	0.035	0.650
**m1 – Class 3**			
	R. t. fennicus ♀	*R. t. fennicus* ♂	*R. t. tarandus* ♀
R. t. fennicus ♂	0.774	-	-
R. t. tarandus ♀	0.774	0.774	-
R. t. tarandus ♂	0.774	0.774	0.774
**m2 – Class 1**			
	R. t. fennicus ♀	*R. t. fennicus* ♂	*R. t. tarandus* ♀
R. t. fennicus ♂	0.955	-	-
R. t. tarandus ♀	**4.594e-03**	**4.594e-03**	-
R. t. tarandus ♂	**0.030**	**4.594e-03**	0.991
**m2 – Class 2**			
	R. t. fennicus ♀	*R. t. fennicus* ♂	*R. t. tarandus* ♀
R. t. fennicus ♂	0.187	-	-
R. t. tarandus ♀	**0.032**	**2.458e-03**	-
R. t. tarandus ♂	**0.029**	**2.458e-03**	0.803

Supplementary Table S12. Multi-test comparisons (*P*-values) on shape data for each wear class for m1 and m2 between the different reindeer 'subspecies + sex' (pairwise Wilcoxon rank tests after the 'Benjamini-Hochberg' correction). A significant contribution was considered for *P*-value < 0.05 (**in bold**).

**m1 – Class 1**	*Wild ♀ R. t. fennicus*	*Wild ♂ R. t. fennicus*	*Wild ♀ R. t. tarandus*	*Wild ♂ R. t. tarandus*	*Domestic ♀ R. t. tarandus*
Wild ♂ R. t. fennicus	0.565	-	-	-	-
Wild ♀ R. t. tarandus	**1.012e-03**	**9.336e-04**	-	-	-
Wild ♂ R. t. tarandus	**1.012e-03**	**2.229e-03**	0.713	-	-
Domestic ♀ R. t. tarandus	**6.161e-04**	**6.777e-05**	0.942	0.848	-
Domestic ♂ R. t. tarandus	**6.161e-04**	**6.161e-04**	0.565	0.984	0.713
**m1 – Class 2**	*Wild ♀ R. t. fennicus*	*Wild ♂ R. t. fennicus*	*Wild ♀ R. t. tarandus*	*Wild ♂ R. t. tarandus*	*Domestic ♀ R. t. tarandus*
Wild ♂ R. t. fennicus	0.704	-	-	-	-
Wild ♀ R. t. tarandus	0.704	0.347	-	-	-
Wild ♂ R. t. tarandus	0.953	0.704	0.561	-	-
Domestic ♀ R. t. tarandus	0.062	0.058	0.170	0.067	-
Domestic ♂ R. t. tarandus	**3.306e-03**	**2.389e-03**	**8.254e-03**	**3.052e-03**	0.062
**m1 – Class 3**	*Wild ♀ R. t. fennicus*	*Wild ♂ R. t. fennicus*	*Wild ♀ R. t. tarandus*	*Wild ♂ R. t. tarandus*	*Domestic ♀ R. t. tarandus*
Wild ♂ R. t. fennicus	0.730	-	-	-	-
Wild ♀ R. t. tarandus	0.777	0.670	-	-	-
Wild ♂ R. t. tarandus	0.940	0.806	0.806	-	-
Domestic ♀ R. t. tarandus	0.670	0.940	0.670	0.730	-
Domestic ♂ R. t. tarandus	0.777	0.940	0.670	0.806	0.940
**m2 – Class 1**	*Wild ♀ R. t. fennicus*	*Wild ♂ R. t. fennicus*	*Wild ♀ R. t. tarandus*	*Wild ♂ R. t. tarandus*	*Domestic ♀ R. t. tarandus*
Wild ♂ R. t. fennicus	0.796	-	-	-	-
Wild ♀ R. t. tarandus	**0.015**	**0.015**	-	-	-
Wild ♂ R. t. tarandus	0.180	0.075	0.474	-	-
Domestic ♀ R. t. tarandus	**0.046**	**0.032**	0.774	0.675	-
Domestic ♂ R. t. tarandus	**0.032**	**0.015**	0.478	0.179	0.364
**m2 – Class 2**	*Wild ♀ R. t. fennicus*	*Wild ♂ R. t. fennicus*	*Wild ♀ R. t. tarandus*	*Wild ♂ R. t. tarandus*	*Domestic ♀ R. t. tarandus*
Wild ♂ R. t. fennicus	0.260	-	-	-	-
Wild ♀ R. t. tarandus	0.314	0.104	-	-	-
Wild ♂ R. t. tarandus	0.186	**0.034**	0.908	-	-
Domestic ♀ R. t. tarandus	**0.034**	**4.390e-03**	0.328	0.464	-
Domestic ♂ R. t. tarandus	**0.034**	**0.022**	0.226	0.282	0.324

Supplementary Table S13. Multi-test comparisons (*P*-values) on shape data for each wear class for m1 and m2 between the different reindeer 'status + sex' (pairwise Wilcoxon rank tests after the 'Benjamini-Hochberg' correction). A significant contribution was considered for *P*-value < 0.05 (**in bold**).

- **m1:** For Class 1, the morphological variation along the negative values of the PC1 showed a squarer morphology, with a deeper lingual interlobal groove and a narrower vestibular interlobal groove (Figure 3A). Also, the vestibulo-lingual diameters of the mesial and distal lobes are wider. These features are that were more associated with wild forest reindeer criteria, while the positive values of the PC1 showed a more tapered morphology with a flatter lingual edge, more related to mountain reindeer criteria. The negative values of the PC2 included more characteristic features of tooth wear, notably with more attenuated lingual enamel folds compared to younger individuals (or less worn teeth) along the positive values of the PC2 with a mesiolingual fold between the metaconid and the parastyle, and a lingual cusp fold between the metaconid and the metastylid, well expressed. For Class 2, the morphological variation along the negative values of the PC2 showed a deeper vestibular interlobal groove and a narrower lingual interlobal groove, with attenuated enamel folds on the lingual side, being more representative of wild forest reindeer (Figure 3B). The variation along the PC1 reflected tooth wear, with the enamel folds more attenuated on the lingual side and the vestibulo-lingual diameters of the mesial and distal lobes larger on the negative values of the PC1. Although there are more overlaps, similar variations have also been demonstrated for Class 3, in which the positive values of the PC1 and PC2 expressed wild forest reindeer characters instead (Figure 3C). However, tooth wear seemed to confuse the taxonomic signal more compared to the less worn teeth.
- **m2:** As with Class 1 of m1, Class 1 of m2 expressed more taxonomic variation in the morphospace on the PC1. Despite the overlaps, the distinction between forest reindeer and mountain reindeer was quite apparent (Figure 3D). This results in a more tapered morphology with a larger mesial vestibulo-lingual diameter than distal in mountain reindeer, whereas the mesial and distal vestibulo-lingual diameters are of more similar proportions in forest reindeer, with a mesiolingual fold between the metaconid and the parastyle that is narrower. Tooth wear also seemed to have an impact on morphology with enamel folds well-marked on the lingual side on positive values of the PC2, and more attenuated on negative values. For Class 2, there are significant overlaps (Figure 3E). It showed rather taxonomic discrimination along the PC2 with a more rectangular conformation on positive values – grouping more mountain reindeer – while it was squarer on negative values – grouping most of the forest reindeer. The PC1 presented characteristics more related to wear, with enamel folds on the lingual side more attenuated towards negative values.

The comparison of the average shape (*Consensus*) calculated for each analysis clearly showed the primary impact of wear on the tooth shape (Supplementary Figure S9), whereby the shape variation evolves according to the wear and the same pattern for the m1 as for the m2. The shape is more rectangular and tapered on slightly worn teeth (i.e. Class 1), with more pronounced lingual enamel folds. The more the tooth is worn, the flatter the lingual side becomes, and the enamel folds become less pronounced. With age, the vestibulo-lingual diameters of the lobes widened, giving a squarer conformation in worn teeth.

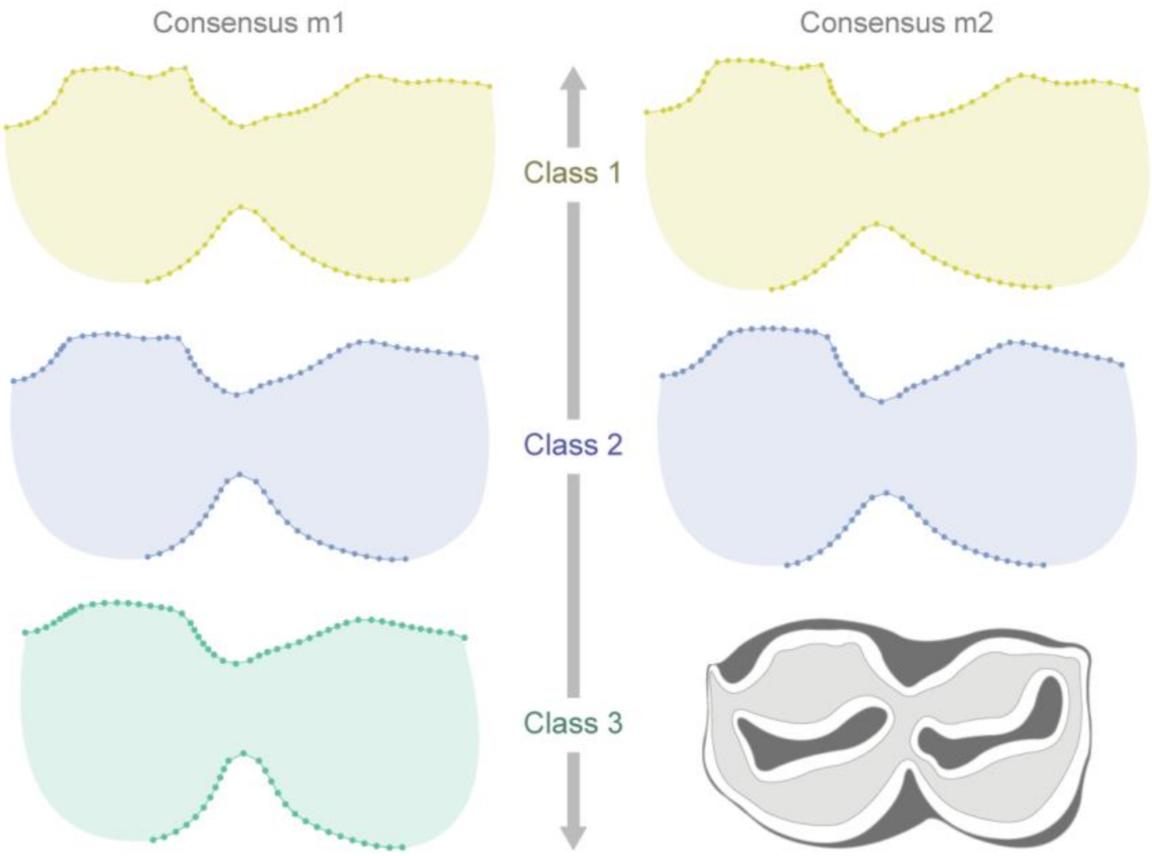

Supplementary Figure S7. Visualisation of the variation of the consensus shape (mean shape) for the m1 and m2 according to the tooth wear class.

6. References

1. Harlin, E., Mannermaa, K. & Ukkonen, P. Animal bones from medieval and early modern Saami settlements in Finnish Lapland in *Helsinki Harvest* (eds. Mannermaa, K., Manninen, M. A., Pesonen, P. & Seppänen, L.) 149-177 (Archaeological Society of Finland, 2019).

2. Salmi, A.-K., van den Berg, M., Niinimäki, S. & Pelletier, M. Earliest archaeological evidence for domesticated reindeer economy among the Sámi of Northeastern Fennoscandia AD 1300 onwards. *J. Anthropol. Archaeol*. **62**, 101303; https://doi.org/10.1016/j.jaa.2021.101303 (2021).

3. Carpelan, C. Juikenttä – näkökulma saamelaiseen yhteiskuntaan. *Oulun yliopisto, Historian laitos, Erikoispainossarja* **287**, 34-44 (1993).

4. Carpelan, C., Jungner, H. & Mejdahl, V. Dating of a sub-recent Saami winter village site near Inari, Finnish Lapland. A preliminary account. *PACT* **36**, 9-26 (1990).

5. Carpelan, C. Peuranpyytäjien talvikylä Inarissa. *Raito* **2**, 20-28 (1991).

6. Carpelan, C. Inarilaisten arkeologiset vaiheet in *Inari – Aanaar. Inarin historia jääkaudesta nykypäivään* (ed. Lehtola, V.) 28-95 (Inarin kunta, 2003).

7. Itkonen, T. I. *Suomen lappalaiset vuoteen 1945* (WSOY, 1948).

8. Korpijaakko, K. *Saamelaisten oikeusasemasta Ruotsi-Suomessa. Oikeushistoriallinen tutkimus Länsi-Pohjan Lapin maankäyttöoloista ja -oikeuksista ennen 1700-luvun puoliväliä*. (Lakimiesliiton kustannus, 1989).

9. Brown, W.A.B. and Chapman N.G. The dentition of red deer (*Cervus elaphus*): a scoring scheme to assess age from wear of the permanent molariform teeth. J. Zool. 224, 519-536; https://doi.org/10.1111/j.1469-7998.1991.tb03783.x (1991).

10. Dudley Furniss-Roe, T. Incremental structures and wear patterns of teeth for ageassessment of Red Deer (BAR International Series 1835, 2008).

11. Jeanjean, M. *et al*. Sorting the flock: Quantitative identification of sheep and goat from isolated third lower molars and mandibles through geometric morphometrics. *J. Archaeol. Sci*. **141**, 105580; https://doi.org/10.1016/j.jas.2022.105580 (2022).

12. Cucchi, T. *et al*. Detecting taxonomic and phylogenetic signals in equid cheek teeth: towards new palaeontological and archaeological proxies. *Royal Soc. Open Sci*. **4**, 160997; http:// doi.org/10.1098/rsos.160997 (2017).

13. Cucchi, T. *et al*. Taxonomic and phylogenetic signals in bovini cheek teeth: Towards new biosystematic markers to explore the history of wild and domestic cattle. *J. Archaeol. Sci*. **109**, 104993; https://doi.org/10.1016/j.jas.2019.104993 (2019).

14. Butler, P. M. The ontogeny of molar pattern. *Biol. Rev*. **31**, 30-69; https://doi.org/10.1111/j.1469-185X.1956.tb01551.x (1956).

15. Korenhof, C. A. W. The enamel-dentine border: a new morphological factor in the study of the (human) molar pattern. *Proc. K. Ned. Akad. Wet*. **64B**, 639-664 (1961).

16. Olejniczak, A. J. *et al*. Morphology of the enamel-dentine junction in sections of anthropoid primate maxillary molars. *J. Hum. Evol*. **53**, 292-301; https://doi.org/10.1016/j.jhevol.2007.04.006 (2007).

17. Skinner, M. M. *et al*. Dental trait expression at the enamel-dentine junction of lower molars in extant and fossil hominoids. *J. Hum. Evol*. **54**, 173-186; https://doi.org/10.1016/j.jhevol.2007.09.012 (2008).

18. Skinner, M. M., Gunz, P., Wood, B. A., Boesch, C. & Hublin J.-J. Discrimination of extant *Pan* species and subspecies using the enamel-dentine junction morphology of lower molars. *Am. J. Phys. Anthropol*. **140**, 234-243; https://doi.org/10.1002/ajpa.21057 (2009).

19. Bailey, S. E., Skinner, M. M. & Hublin, J.-J. What lies beneath? An evaluation of lower molar trigonid crest patterns based on both dentine and enamel expression. *Am. J. Phys. Anthropol*. **145**, 505-518; https://doi.org/10.1002/ajpa.21468 (2011).

20. Ortiz, A., Skinner, M. M., Bailey, S. E. & Hublin, J.-J. Carabelli's trait revisited: an examination of mesiolingual features at the enamel-dentine junction and enamel surface of *Pan* and *Homo sapiens* upper molars. *J. Hum. Evol*. **63**, 586-596; https://doi.org/10.1016/j.jhevol.2012.06.003 (2012).

21. Rohlf, F. J. *tpsDig* (Stony Brook, 2010).

22. Prat, F. Quelques mammifères pléistocènes. Odontologie, ostéologie. Université Bordeaux 1, Institut du Quaternaire, mimeograph course, 245 p. (1970-75).
